# SARS‐CoV‐2 Spike Protein‐Derived Cyclic Peptides as Modulators of Spike Interaction with GRP78

**DOI:** 10.1002/cbic.202300789

**Published:** 2024-05-29

**Authors:** Nicholas Johnson, Craig Pattinson, Kate Burgoyne, Karolin Hijazi, Wael E. Houssen, Bruce F. Milne

**Affiliations:** ^1^ Institute of Medical Sciences University of Aberdeen Ashgrove Road West Aberdeen AB25 2ZD UK; ^2^ School of Medicine Medical Sciences and Nutrition University of Aberdeen Aberdeen AB25 2ZD UK; ^3^ Department of Chemistry University of Aberdeen Meston Walk Aberdeen AB24 3UE UK; ^4^ CFisUC Department of Physics University of Coimbra Rua Larga 3004-516 Coimbra Portugal

**Keywords:** BiP, Cyclic peptides, GRP78, HSPA5, SARS-CoV-2

## Abstract

The human glucose‐regulated protein GRP78 is a human chaperone that translocactes to the cell surface when cells are under stress. Theoretical studies suggested it could be involved in SARS‐CoV‐2 virus entry to cells. In this work, we used in vitro surface plasmon resonance‐based assays to show that human GRP78 indeed binds to SARS‐CoV‐2 spike protein. We have designed and synthesised cyclic peptides based on the loop structure of amino acids 480–488 of the SARS‐CoV‐2 spike protein S1 domain from the Wuhan and Omicron variants and showed that both peptides bind to GRP78. Consistent with the greater infectiousness of the Omicron variant, the Omicron‐derived peptide displays slower dissociation from the target protein. Both peptides significantly inhibit the binding of wild‐type S1 protein to the human protein GRP78 suggesting that further development of these cyclic peptide motifs may provide a viable route to novel anti‐SARS‐CoV‐2 agents.

## Graphical abstract

The chaperone protein GRP78 mediates cell entry by various pathogens, including SARS‐CoV‐2. Cyclic peptides based on surface features of SARS‐CoV‐2 spike protein were synthesised and found to inhibit spike interaction with GRP78. Wuhan and Omicron spike sequences were compared with the known GRP78 ligand Pep42, with the Omicron‐derived peptide showing the highest activity.

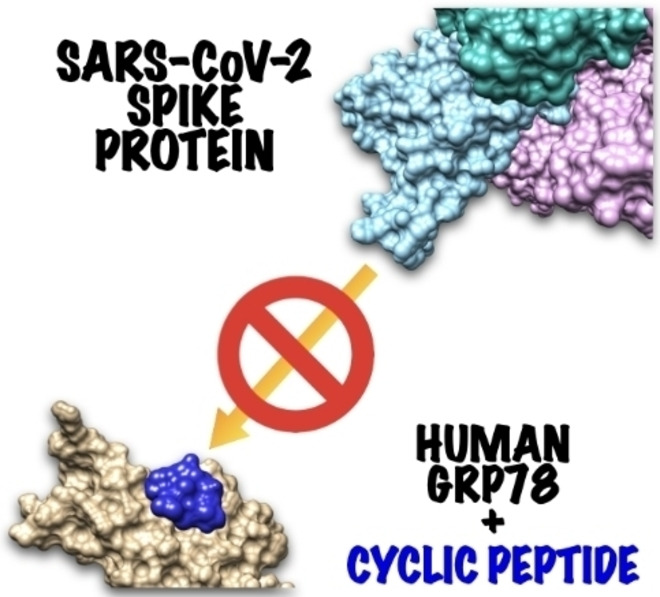

## Introduction

The 78 kDa chaperone glucose‐regulated protein GRP78 (also known as heat shock protein A5 (HSPA5) or binding immunoglobulin protein (BiP)) is expressed mainly in the endoplasmic reticulum of human cells where it has a vital role in managing the correct folding of nascent proteins.[^1^, ^2^] In cells undergoing stress such as that encountered in disease states such as cancer and infection, GRP78 is upregulated and translocates to the cell surface (csGRP78).[^3^, ^4^, ^5^]

The increased expression of csGRP78 in cancer has been the focus of previous work aimed at using the protein to target tumour cells with compounds bearing apoptosis‐inducing warheads. In particular, a 13‐mer cyclopeptide, Pep42, was found to display strong and specific binding to csGRP78 and was able to induce internalization of both fluorescent markers and anti‐cancer drugs upon complexation with csGRP78.[^6^, ^7^, ^8^]

A number of important viral pathogens take advantage of cs‐GRP78 to facilitate entry into the cell. These include Flaviviruses (e. g., Zika,[^9^, ^10^] Dengue,[^1^, ^11^, ^12^] West‐Nile and Japanese encephalitis virus (JEV)^[13]^ as well as coronaviruses (MERS, and HKU9)^[14]^ and enteroviruses (Coxsackie virus A9).^[15]^ Because of csGRP78’s importance in mediating cell entry for potentially lethal viruses, a significant body of work has been built up in the literature concerning the details of the interactions between viral surface proteins and csGRP78 and it has been shown in‐vitro that blocking this interaction with csGRP78‐specific antibodies can prevent entry of Zika, JEV and SARS‐CoV‐2 into cells.[^10^, ^13^, ^16^]

Theoretical studies suggested that binding between the SARS‐CoV‐2 spike protein (SP) and csGRP78 involves a 9‐residue disulphide‐bridged loop spanning residues 480–488 on the SP surface.[^17^, ^18^, ^19^] Similar sequences/loops exist in a number of other spike/envelope proteins from other viruses^[17]^ and are known to interact with csGRP78 suggesting that these loops may be important starting points for the development of CP drugs that can specifically target the relevant binding site on csGRP78 and act as competitive inhibitors to prevent cell entry in a broad range of important viral diseases.^[20]^

In the current manuscript, we report the computational design and protein‐ligand docking of two loop‐derived cyclic peptides (LCP) based on the disulphide‐bridged S1 surface loops found in the Wuhan (LCP_*W*_) and Omicron (LCP_*O*_) strains along with the known GRP78 binder, Pep42. Based on the results of this in‐silico work, the synthesis and experimental evaluation of the binding of these peptides to GRP78 have been performed.

## Results and Discussion

### Protein‐Ligand Docking

**Figure 1:**
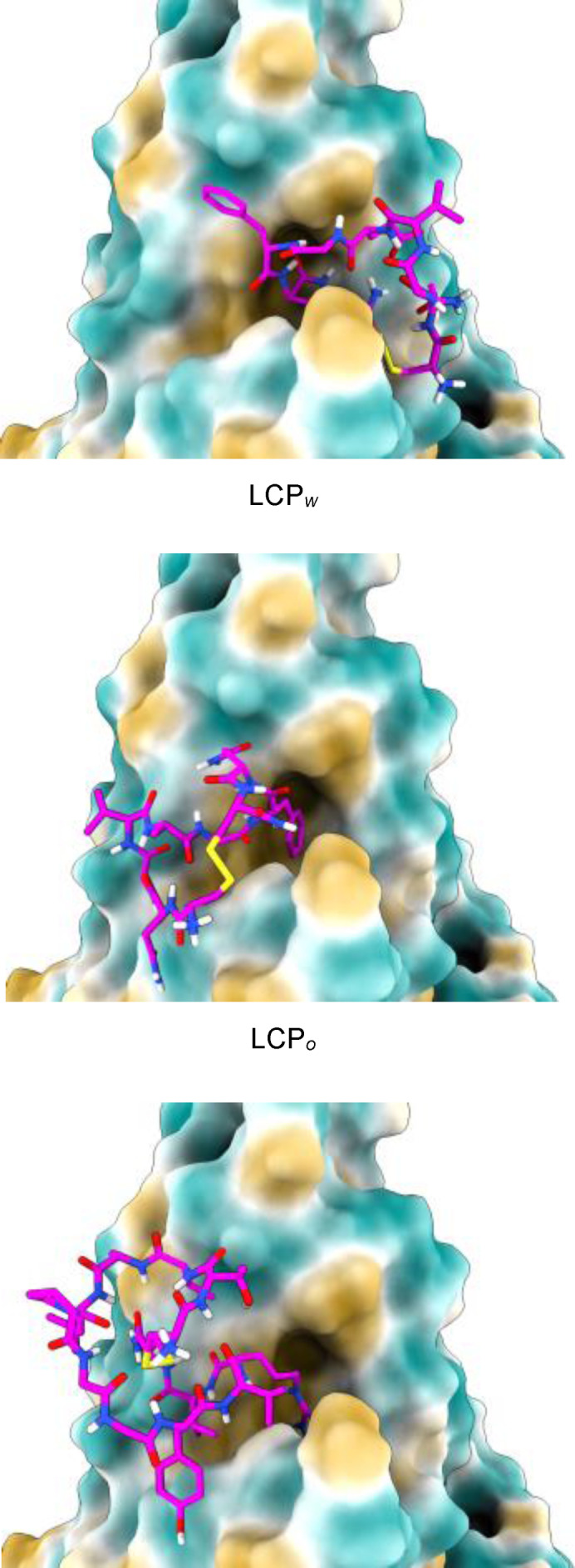
GRP78 SBD*β* with docked cyclic peptides; LCP_*w*_ (top panel), LCP_*o*_ (middle panel) and Pep42 (lower panel). Images show the previously identified SARS‐CoV‐2 spike interaction region (IV).^[17]^ Protein surface coloured by hydrophilicity: blue=hydrophilic, yellow=hydrophobic.

The sequences of the synthesized three peptides are shown in Table 1. The best docking poses obtained for the three peptides are shown in Figure 1. In this figure, the protein surface is coloured by hydrophobicity, with yellow areas being the most nonpolar and blue areas being the most polar. It can be seen that all of the peptides interact with a deep hydrophobic pocket in the center of the substrate binding domain (SBD*β*). This pocket forms part of the region previously predicted to be involved in the spike : GRP78 interaction using very different protein‐protein docking methods and for this reason it is interesting that our calculations also highlight this region.^[17]^

**Table 1:** Synthetic peptides used in this study with best scores from docking calculations.

Peptide	Sequence	Docking Score (kcal mol^−1^)
LCP_*W*_	Cyclo (NH_2_−C*NGVEGFNC*−CONH_2_)	−7.2
LCP_*O*_	Cyclo (NH_2_−C*NGVAGFNC*−CONH_2_)	−7.8
Pep42	Cyclo (NH_2_−C*TVALPGGYVRVC*−CONH_2_)	−6.7

The fact that the LCP_*W*_ and LCP_*O*_ peptides bind to different faces of SBD*β* might support a difference in the binding mode of the spike surface loops from the Wuhan and Omicron variants to this region of GRP78. This would be consistent with alterations in the binding of the variants’ spike proteins to GRP78 and may go some way to rationalizing differences in their infectivity, given that only reasonably small sequence differences are seen in the two spike proteins. The top ten docking poses for LCP_*W*_ and LCP_*O*_ are shown in Figures S37 and S38, respectively. In addition to displaying a lower scoring best pose, LCP_*O*_ can be seen to bind only at the hydrophobic pocket site, whilst LCP_*W*_ binds both at this site and also in a completely different region of the SBD*β* domain. This and the fact that there is much greater diversity in the details of the binding modes, suggest that LCP_*W*_ is a much more promiscuous binder, which is in line with the experimental Biacore results, presented below. It can also be seen from the SI figures, that despite LCP_*O*_ binding on the left of the hydrophobic pocket in its best pose, there is a clear consensus from the other nine poses for the favoured binding site to be on the right of the image, which is in line with the previous binding site prediction of Ibrahim et al.^[17]^ Furthermore, it was noted that for LCP_*O*_ all ten poses have the Phe side chain inserted into the hydrophobic pocket, whilst only half of the predicted poses for LCP_*W*_ displayed this binding feature, regardless of whether they were close to the hydrophobic pocket or not. A list of the GRP78 SBD*β* residues involved in the predicted binding of LCP_*W*_ and LCP_*O*_ is given in Table S3. From this list, it can be seen that the hydrophobic pocket constitutes the majority of the list of residues identified by Ibrahim et al.^[17]^

The reference peptide Pep42, which was designed to be an efficient GRP78 ligand, was predicted to bind to the same region of GRP78 SBD*β* as LCP_*O*_. Assuming that the Wuhan and Omicron spike loops do bind in a manner similar to that shown by the synthetic loop peptides, this also suggests better binding by the Omicron loop due to structural differences caused by the E484 A mutation.

The binding scores corresponding to the poses in Figure 1 are shown in Table 1. Although Pep42 and LCP_*O*_ are predicted to bind to the same region of SBD*β*, the loop peptide is predicted to bind more strongly, suggesting that LCP_*O*_ should be a better ligand for this region of GRP78 than the known ligand Pep42. In addition, the score for LCP_*W*_ is less than that of LCP_*O*_, again suggesting that the Omicron‐derived peptide should display the strongest binding. The differences in the binding strengths predicted by the docking scores appear to correlate with the way in which the peptides interact with the hydrophobic pocket in the SDB*β* structure. Whilst all three peptides display some degree of interaction, LCP_*O*_ is the only peptide which inserts a nonpolar Phe side chain into this pocket. Although LCP_*W*_ also possesses this side chain, the conformation of the docked pose for this peptide means that the Phe side chain is directed out of the hydrophobic pocket. In the poses observed for the other two peptides, the groups interacting with the pocket are relatively polar, which would lead to a less favorable binding interaction.

The large size of the ligands means that this can only be a partial explanation, due to the many complimentary/competing protein‐ligand interactions that are occurring. However, the fact that this pocket features prominently in both our in silico results and in the methodologically very different protein‐protein docking study of Ibrahim et al, suggests that this is a key interaction site.^[17]^

### Binding of Spike Protein to GRP78

**Figure 2:**
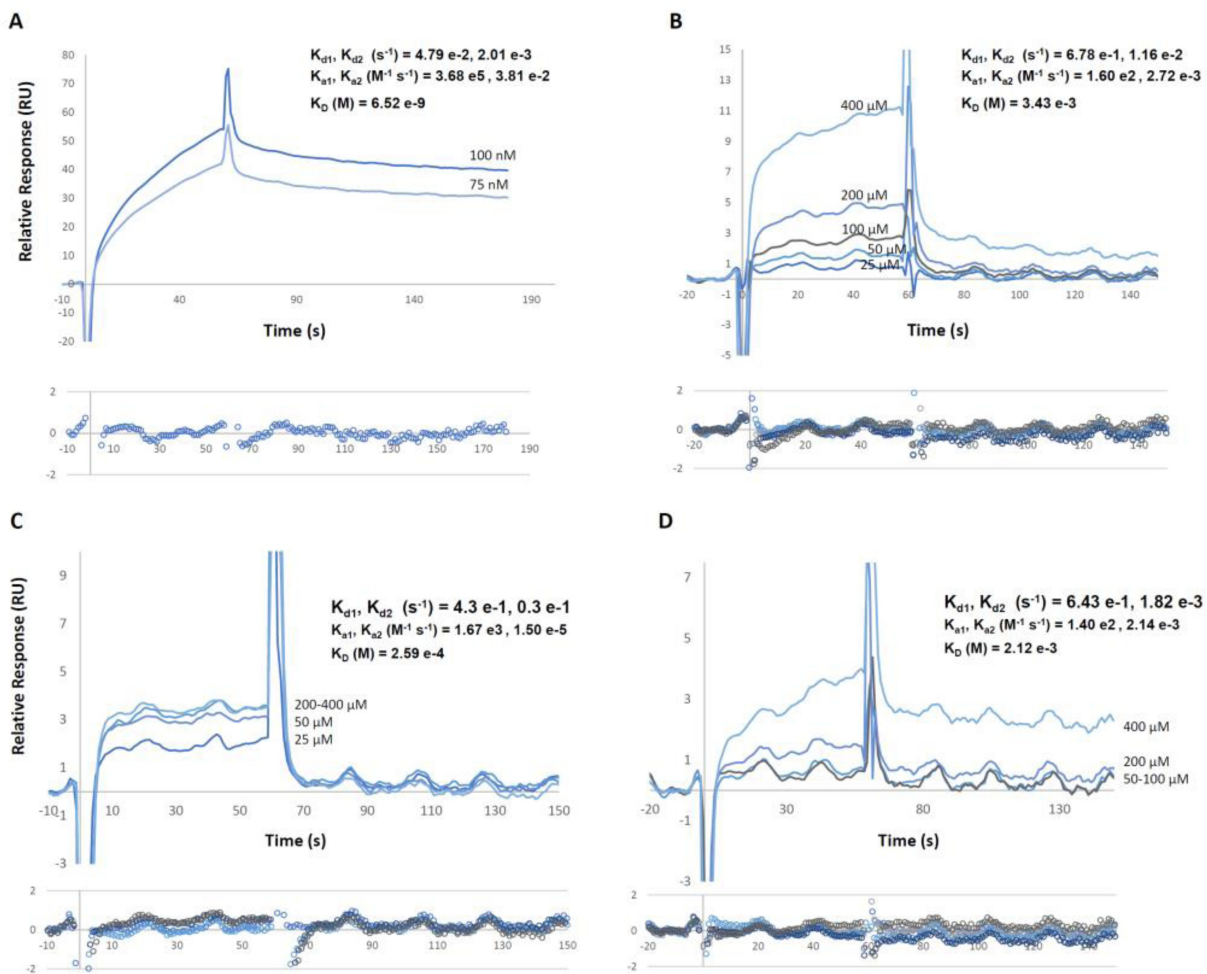
Binding activity to GRP78. (A) Superimposed sensorgrams representing binding activity of S1 to immobilised GRP78. (B–D) Superimposed sensorgrams representing binding activity of fluid phase reference peptide Pep42 and two spike loop peptides to immobilised GRP78. Equilibrium dissociation constants (KD) as well as association (ka) and dissociation constant (kd) rates are inset. Distribution of residuals for sensorgrams fitted to a 2‐state reaction binding model is shown beneath each plot.

Direct binding activity between GRP78 and the spike protein of SARS‐CoV‐2 was demonstrated in two surface plasmon resonance assay configurations. Firstly, fluid phase soluble S1 was injected on immobilized GRP78. S1 showed dose‐dependent binding to GRP78 with the following kinetics parameters as determined by sensorgram fitting to the 2‐state reaction model: *K*_*d*1_, *K*_*d*2_ (s^−1^)=4.79×10^−2^, 2.01×10^−3^; *K*_*a*1_, *K*_*a*2_ (M^−1^ s^−1^)=3.68×10^5^, 3.81×10^−2^; *K_D_* =6.52×10^−9^ M (Figure 2A). The 2‐state reaction model describes a 1 : 1 binding to the immobilised ligand followed by a conformational change that stabilises the complex. In the reverse configuration where fluid phase soluble GRP78 was injected on immobilized S1, kinetics parameters differed with an overall lower affinity: *K*_*d*1_, *K_d2_* (s^−1^)=7.66×10^−2^, 1.58×10^−3^; *K*_*a*1_, *K*_*a*2_ (M^−1^ s^−1^)=3.28×10^4^, 5.23×10^−2^; *K_D_* =6.86×10^−8^ M (Data not shown).

Fluid phase spike loop and reference peptides were injected on immobilized GRP78. The LCP_*W*_ and LCP_*O*_ loop peptides, and the reference peptide Pep42 showed dose‐dependent binding to GRP78 with affinities of 0.2 μM, 2 μM and 3.4 μM, respectively as determined by sensorgram fitting to the 2‐state reaction model. The binding activity of the two spike loop peptides and Pep42 to GRP78 is shown in Figure 2 B–D. LCP_*W*_, showed the strongest binding affinity (K_D_=2×10^−4^ M). However, this peptide showed a faster final dissociation constant rate (K_d2_=0.3×10^−1^ s^−1^) compared to LCP_*O*_ (K_d2_=1.82×10^−3^ s^−1^), suggesting higher interaction stability of the Omicron LCP with GRP78 compared to the Wuhan derivative (Figure 2 C–D).

### Spike Loop Peptides as Antagonists of SARS‐CoV‐2 Spike

Surface plasmon resonance competition assays were used to evaluate the inhibitory properties of the spike loop peptides on S1 binding to GRP78. Inhibition assays were carried out by immobilization of S1 on the sensorchip surface and binding measurement of fluid phase GRP78 at a fixed concentration in presence of spike loop peptides or the reference peptide Pep42.

**Figure 3:**
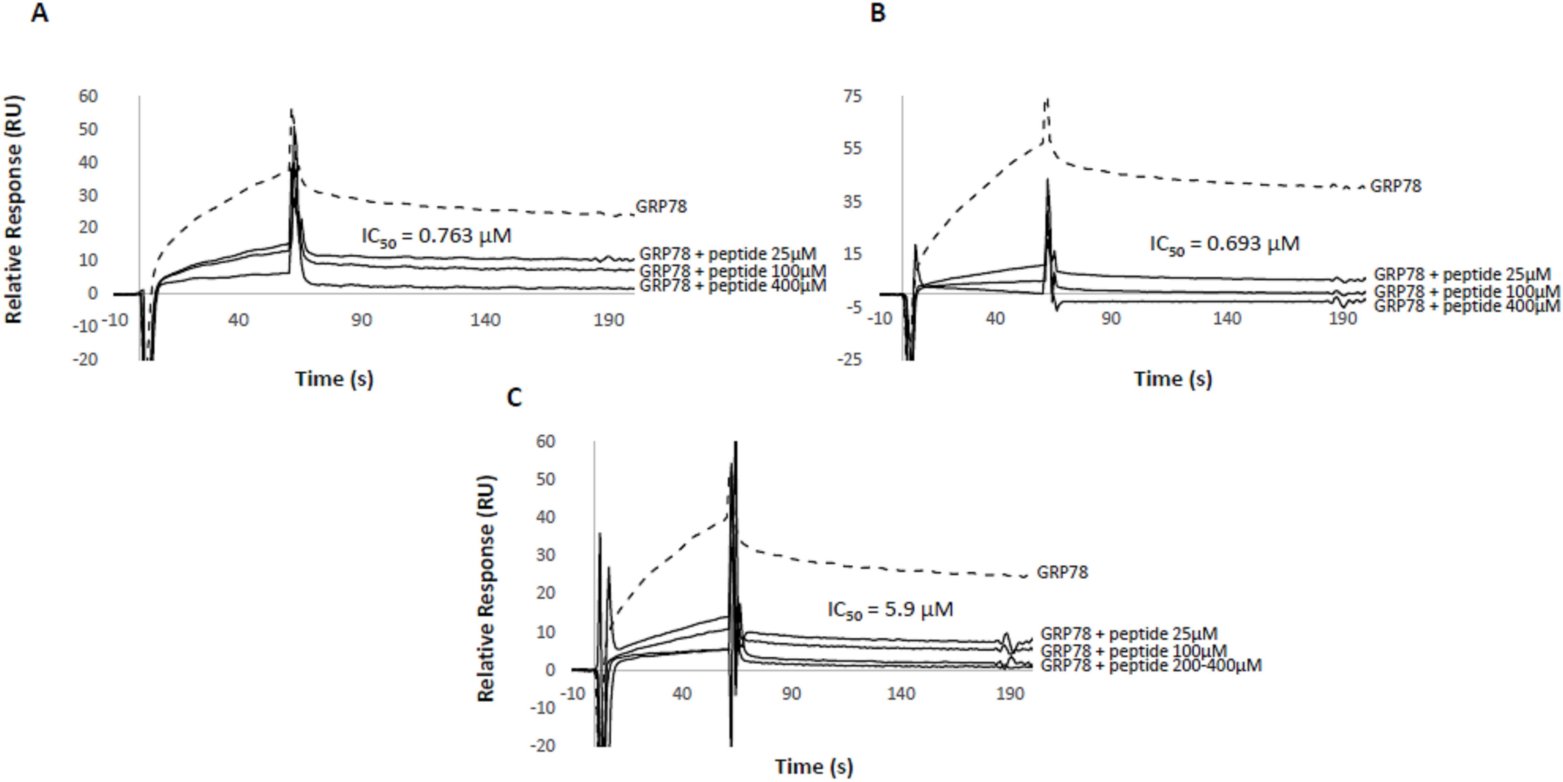
Inhibitory activity of spike loop peptides. S1 subunit of the spike protein was immobilized on the sensorchip by amine coupling; binding activity of fluid phase GRP78 in presence of peptides was determined and compared to GRP78 alone. Superimposed sensorgrams representing GRP78 (500 nM) binding activity in absence (dotted curve) and presence (solid curves) of Wuhan loop peptide LCP_*W*_ (A), Omicron loop peptide LCP_*O*_ (B), Pep42 (C). IC_50_ values estimated by logistic regression are inset.

The two spike loop peptides inhibited GRP78:S1 interaction to a similar extent and with IC_50_ values in the sub‐micromolar range. As shown in Figure 3 A–C, IC_50_ values for the Omicron and Wuhan peptides were 693 nM and 763 nM respectively and lower than the IC_50_ value observed for the reference peptide Pep42 (5.90 μM). GRP78 binding to immobilised S1 in the presence of lower concentrations of the Omicron and Wuhan peptides (12.5 μM) showed lower binding affinities (K_D_ of 7.51×10^−7^ M and 1.37×10^−7^ M, respectively; data not shown) compared to binding of GRP78 alone (K_D_=6.86×10^−8^ M) suggesting a destabilizing effect by the peptides (LCP_*O*_>LCP_*W*_) on the GRP78 : S1 interaction at this low concentration.

## Conclusions

Based on the results shown above, we conclude that SARS‐CoV‐2 spike protein can bind to human GRP78 and that the SP surface loop spanning residues 480–488 is the key motif for this interaction. This interaction may provide a route for virus entry to cells. Peptides designed based on the structure of this loop could inhibit the interaction between spike protein and GRP78 and are good starting point to design inhibitors for this target.

## Experimental Section

### Computational

Unless otherwise stated, default values for software settings were used in the methods, below.

**Generation of conformational ensembles**: Ensembles for the three cyclic peptides were generated using the iterative meta‐dynamics with genetic crossing (iMTD‐GC) method implemented in the CREST software package (version 2.10) interfaced to the extended tight‐binding semi‐empirical electronic structure code ×TB (version 6.4.0).[^21^, ^22^] The underlying electronic structure method used in the CREST calculations was the self‐consistent‐charge tight‐binding model GFN2‐×TB which includes the recent D4 density‐dependent dispersion correction.[^23^, ^24^] The generalised Born surface‐area (GB/SA) continuum solvent model for water was used for all CREST/×TB calculations. Due to the large size of the Pep42 peptide, the metadynamics simulations were prohibitively large, given the available computational resources. For this reason, the iMTD‐GC calculations were run with the faster GFN‐FF method. The resulting conformational ensemble was then re‐optimised and reordered at the GFN2‐×TB level, to give the final ensemble for use in the docking calculations.

All local minima found up to a cutoff of 25 kJ/mol were saved and formed the final ensembles used in the docking calculations.

**Protein**‐**ligand docking**: Docking calculations were performed using the Autodock Vina software package and scoring function (version 1.1.2).^[25]^ Preparation of the target protein structures as well as placement and sizing of the simulation box was performed in UCSF Chimera (version 1.16).^[26]^

For the docking to GRP78, a 40×40×40 Å simulation box (shown in Figure S36) centred on Ile459 in the middle of the GRP78 substrate‐binding domain β (SBD*β*) was used. Ile459 was chosen because it lies in the centre of region IV of the SBDβ which was previously predicted to be the binding site of the spike 480–488 loop.^[17]^ The size of the box was chosen to encompass the whole of the GRP78 SBDβ domain, in order to avoid biasing the calculations towards docking solutions that favoured the previously predicted binding site. For each ensemble member, the docking was performed to obtain the single best‐scoring pose. The exhaustiveness parameter in the Vina input was increased from its default value of 8 to 32, to provide more complete conformational searching during the docking pose generation.

The coordinates of the GRP78 protein (accession code 5E84) were obtained from the Protein Databank.^[27]^ This structure was used in the original computational investigation of spike/GRP78 binding and was chosen here to be consistent with the previous work.^[17]^

Chain A from the PDB structure file was used in the docking work presented here. The various members of the conformational ensembles were docked against the 5E84 structure using the database screening script obtained from the Autodock Vina website at https://vina.scripps.edu/wp‐content/uploads/sites/55/2020/12/vina_screen_local.sh.

### Chemical Synthesis

**Solid Phase Peptide Synthesis**: Fmoc‐Ala‐OH, Fmoc‐Asn (Trt)‐OH, Fmoc‐Cys (Trt)‐OH, Fmoc‐Glu (OtBu)‐OH, Fmoc‐Gly‐OH, Fmoc‐Phe‐OH, Fmoc‐Pro‐OH, Fmoc‐Thr (OtBu)‐OH, and Fmoc‐Val‐OH were purchased from Novabiochem, Merck Biosciences, UK. Fmoc‐Leu‐OH, N,N’‐diisopropylcarbodiimide (DIC)), trifluoroacetic acid (TFA), and triisopropylsilane (TIS) were acquired from Fluorochem, UK. Fmoc‐Arg(Pbf)‐OH, Fmoc‐Tyr(tBu)‐OH, ethyl cyano(hydroxyimino)acetate (Oxyma Pure), and the Fmoc‐Rink Amide ProTide (LL) resin were obtained from the CEM corporation, USA. N,N‐dimethylformamide (DMF), dichloromethane (DCM) and diethyl ether were purchased from VWR, Avantor, USA. Piperidine was purchased from Merck Life Sciences, UK. and 2,2’‐(ethylenedioxy) diethanethiol (DODT), dimethyl sulfoxide (DMSO), and ammonium carbonate were purchased from Sigma Aldrich, UK. 100 % acetic acid was purchased from BDH, UK.

Linear precursor peptides were prepared using the standard Fmoc‐based solid‐phase peptide synthesis (SPPS) strategy on a Liberty BlueTM Automated Microwave Peptide Synthesizer (CEM Corporation, USA) at a 0.1 mmol scale. Fmoc‐Rink Amide Pro‐Tide (LL) resin (0.18 mmol/g) was used for the synthesis of all the peptides. Initial deprotection of the Fmoc protecting group from the resin and all subsequent deprotections were performed with programmed cycles using a solution of 20 % piperidine in DMF. Coupling cycles in the synthesizer were performed, after deprotection, with solutions of 0.2 M Fmoc‐amino acids (5 eq), 1 M DIC (10 eq) and 1 M Oxyma (10 eq) in DMF. Most Fmoc amino acids underwent one coupling cycle at 90 °C during attachment. Fmoc‐Arg (Pbf)‐OH residues underwent two coupling cycles at 75 °C before deprotection and Fmoc‐Cys (Trt)‐OH underwent a single coupling cycle set at 50 °C. For all peptides, the N‐terminal Fmoc group was cleaved at the end of the synthesis. After synthesis, the peptide resin was transferred to an empty fritted SPE cartridge, rinsed with DCM (6×), and dried under vacuum.

The peptides were cleaved from the resin by treatment with a cocktail solution of TFA/TIS/DODT/H_2_O (92.5 : 2.5 : 2.5 : 2.5) for 3 h. at RT on an orbital shaker (Heidolph, 1350 rpm). The cleavage mixture was then concentrated under a stream of N_2_ gas. The peptides were precipitated using cold diethyl ether, placed into a −20 °C freezer overnight, washed with ether (3×) and dried under vacuum to give the crude solid.

**Disulphide Bridge Cyclisation**: The crude peptide solids were cyclised through a disulphide bridge connecting the two terminal cysteines. The bridge was formed through a standard oxidation reaction with the peptide and DMSO under dilute conditions.^1^ The cleaved peptide was dissolved into a solution of 10 % acetic acid in water (1 mg/mL). The pH of the solution was increased to 6 through the addition of solid ammonium carbonate. Once at pH 6, DMSO was added to the solution to make it 10 % by volume and was left to stir at RT overnight. The progress of the reaction was monitored using LCMS.^2^ Once the reaction was completed, the DMSO was diluted with the addition of water and the solution was lyophilized on the LaboGene CoolSafe Freeze dryer to give the crude solid.

**Peptide Purification**: The oxidised peptides were purified out using reversed phase HPLC on an Agilent Technologies 1260 Infinity system using a C18 column (ACE 5 C18‐HL, 5 μm, 10×250 mm, 100 Å) through an Acetonitrile (+0.1 % TFA)/Water (+0.1 % TFA) gradient. The collected fractions were subsequently lyophilized to give the pure solid. Identity and purity were confirmed by HPLC‐MS and NMR spectra analyses .^3^

### Assay

**Recombinant Proteins**: The S1 subunit (aa 14–685) and the receptor binding domain (RBD) of the spike protein of SARS‐CoV‐2 (aa 319–541) were recombinantly produced in HEK293 cells and provided by Peak Proteins Ltd (Macclesfield, UK). The full length human GRP78 protein recombinantly produced in Escherichia coli was obtained from Abcam (Cambridge, UK).

**Surface Plasmon Resonance Assays**: Surface plasmon resonance experiments using BIAcore (Cytiva, Uppsala, Sweden) were carried out to measure i) the direct interaction of GRP78 with the SARS‐CoV‐2 spike protein as well as the synthetic peptides mimicking the spike loop predicted to interact with GRP78 and ii) antagonistic competition for GRP78 interaction by the peptides versus the S1 subunit of the spike protein.

**Affinity Measurements**: GRP78 in PBS−P+ buffer (0.2 M phosphate buffer, 27 mM KCl and 1.37 M NaCl, 0.5 % surfactant P20, pH 7.4) supplemented with EDTA 50 μM was immobilized (approximately 1800 RU) on the surface of flow cell 2 of an NTA sensorchip using the standard nickel activation procedure (Cytiva). This was followed by activation with a mixture of 1‐ethyl‐3‐(3‐ dimethylaminopropyl) carbodiimide (EDC) and N‐hydroxysuccinimide (NHS) for 7 minutes and inactivation with ethanolamine for 7 minutes for covalent linking of the 6×His‐tag‐RBD to the nickel‐activated surface. Flow cell 1 (treated with the same reagents except for GRP78) served as a reference (blank) cell. Binding of synthetic peptides LCP_*W*_, LCP_*O*_ and Pep42 with the recombinant S1 of SARS‐CoV‐2 was determined at 2‐fold serial concentrations ranging from 25 μM to 400 μM. Binding of fluid phase S1 was measured at concentrations <75 nM. Peptides and S1 were dissolved in PBS−P+ buffer supplemented with 5 % DMSO. Contact time was 60 seconds and flow rate was 30 μL/min. The surface was regenerated with 350 mM EDTA, followed by a further regeneration with 0.5 % (w/v) SDS. Equilibrium dissociation constants (*K_D_*) as well as association (*k_a_*) and dissociation constant (*k_d_*) rates were calculated using the Biacore×100 Evaluation software version 2.0.2 (Cytiva). Curves were first fitted to pre‐defined binding models selecting that which gives best fit as judged by the lowest χ2 value and best distribution of residuals.

**Inhibition Studies**: *GRP78* : *S1 inhibition*: Recombinant S1 in 10 mM sodium acetate pH 5.0 was immobilized (approximately 2100 RU) on the surface of flow cell 2 of a CM5 sensorchip using standard amine coupling. Flow cell 1 (treated with the same reagents except for S1) served as a reference (blank) cell. Binding of fluid phase GRP78 at a concentration of 500 nM was determined in the presence of peptides at 3 μM–400 μM and compared to binding activity of GRP78 alone. Background sensorgrams of the peptides alone to S1 were subtracted from the sensorgrams generated by injection of peptides in presence of soluble GRP78. Fluid phase GRP78 and peptides were dissolved in HBS‐EP+ buffer (0.1 M HEPES, 1.5 M NaCl, 0.03 M EDTA, 0.5 % surfactant P20, pH 7.4) supplemented with 5 % DMSO. Flow rate was 30 μL/min and contact time was 60 seconds. After each measurement, the surface was regenerated with 10 mM glycine‐HCl pH 1.5.

The half‐maximal inhibitory concentration (IC_50_) was defined as the concentration of peptide that reduced GRP78 binding to S1 or RBD to 50 % when compared with binding of GRP78 alone. IC50 values were calculated by logistic logarithmic regression using Microsoft Excel. All conditions were assessed in two independent experiments.

## Conflict of interests

The authors declare no conflict of interest.

1

## Supporting information

As a service to our authors and readers, this journal provides supporting information supplied by the authors. Such materials are peer reviewed and may be re‐organized for online delivery, but are not copy‐edited or typeset. Technical support issues arising from supporting information (other than missing files) should be addressed to the authors.

Supporting Information

## Data Availability

The data that support the findings of this study are available in the supplementary material of this article.

## Footnotes

n1 Merck, Protocols for the Fmoc SPPS of Cysteine‐containing Peptides, https://www.sigmaaldrich.com/GB/en/technical‐documents/protocol/chemistryand‐synthesis/peptide‐synthesis/fmoc‐spps‐cysteine‐peptides#synthesis, (accessed April 14, 2022).

n2 ESI‐MS (Agilent 1200 equipped with Agilent 6120 Quadrupole ESI‐MS on an ACE 5C18‐HL column (5 μm, 250×4.6 mm id, 100 A). A mobile phase of 5 % ACN (+0.1 % TFA) to 100 % ACN (+0.1 % TFA) was used).

n3 Agilent 1290 UHPLC coupled to a Bruker MAXIS II Q‐ToF mass spectrometer. For LC, A Kinetex 2.6 μM 100×2.1 mm EVO C18 100 A column was used. A mobile phase of 5 % ACN (+0.1 % formic acid) to 100 % ACN (+0.1 % formic acid) was used. Mass range was set to m/z 100–2000, capillary voltage 4.5 kV, nebular gas 4.0 bar, dry gas 9.0 L/min, and dry temp to 250 °C. MS/MS experiments were conducted under Auto MS/MS scan mode with a step collision energy from 80–200 %.
