# Supplementary material for: SARS‐CoV‐2 Spike Protein‐Derived Cyclic Peptides as Modulators of Spike Interaction with GRP78
Source: Chembiochem. 2024 May 29;25(12):e202300789. doi: 10.1002/cbic.202300789 (PMC11497264; doi:10.1002/cbic.202300789)
Supplement: Supplementary file 1 — Supporting Information [file CBIC-25-e202300789-s001.pdf]

ChemBioChem

Supporting Information

SARS-CoV-2 Spike Protein-Derived Cyclic Peptides as Modulators of Spike Interaction with GRP78

Nicholas Johnson, Craig Pattinson, Kate Burgoyne, Karolin Hijazi,* Wael E. Houssen,* and Bruce F. Milne*

Supporting Information

SARS-CoV-2 spike protein-derived cyclic peptides as modulators of spike interaction with GRP78

Nicholas Johnson, Craig Pattinson, Kate Burgoyne, Karolin Hijazi, Wael E. Houssen and Bruce F. Milne

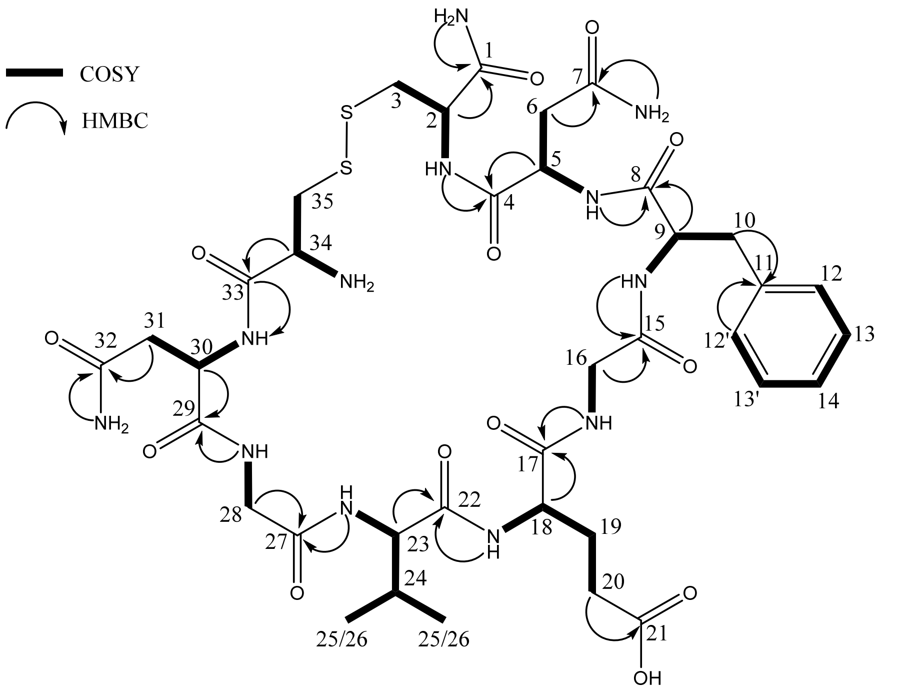

Figure S1: Chemical structure of LCP_W shown with structural assignment numbering based on Table S1. ^1H-^1H COSY correlations are shown as thick bonds and ^1H-^{13}C HMBC correlations as arrows.

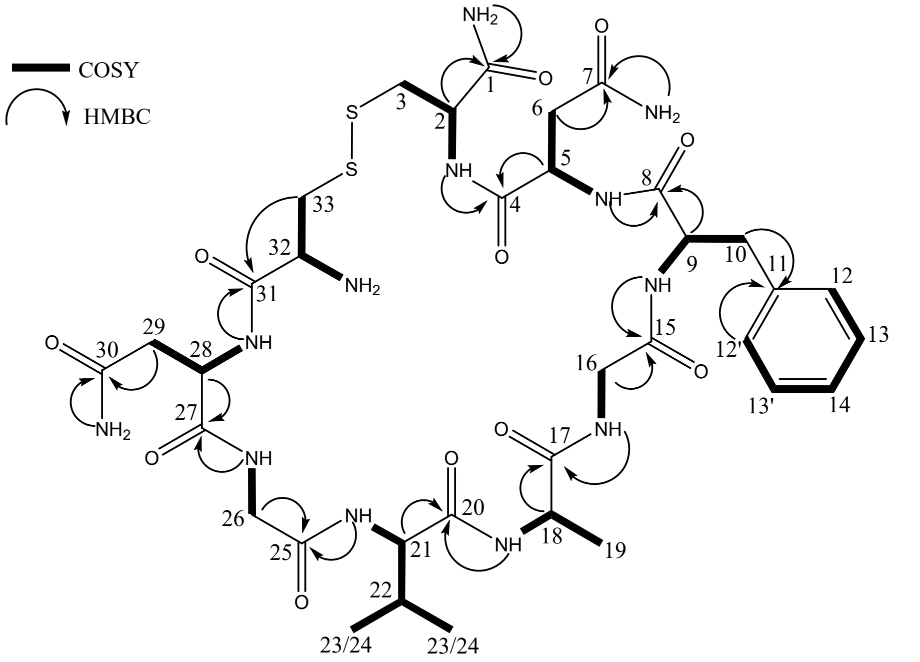

Figure S2: Chemical structure of LCP_O shown with structural assignment numbering based on Table S2. ^1H-^1H COSY correlations are shown as thick bonds and ^1H-^{13}C HMBC correlations as arrows.

Table S1: ^{13}C NMR (100 MHz) and ^{1}H (400 MHz) NMR data for LCP_W in DMSO-d_6. ^{13}C values from carbon and HSQC spectra and ^{1}H values from proton and HSQC spectra.

Residue	Pos.	$^{13}C\delta$ (ppm)	C/N-Type	$^{1}H\delta$ (ppm) (Proton count, m, J (Hz))	HMBC Data ($^{13}C \rightarrow {}^{1}H$)
Cys (1)	1	171.57	C	-	H-2, H-3, H-2-NH, H-1-NH_2
	2	52.33	CH	4.45 (1H, *m*)	H-3, H-2-NH
	3	41.15	CH_2	A: 3.13 (1H, *m*)<br>B: 2.99 (1H, *m*)	H-2
	2-NH	-	NH	8.15 (1H, *m*)	-
	1-NH_2	-	NH_2	A: 7.48 (1H, *s*)<br>B: 7.32 (1H, *s*)	-
Asn (1)	4	171.26	C	-	H-2, H-5, H-6, H-2-NH, H-5-NH,
	5	50.31	CH	4.54 (1H, *m*)	H-6, H-5-NH
	6	36.66	CH_2	A: 2.62 (1H, *m*)<br>B: 2.50 (1H, *m)*	H-5, H-7-NH_2
	7	172.37	C	-	H-5, H-6, H-7-NH_2
	5-NH	-	NH	8.40 (1H, *m*)	-
	7-NH_2	-	NH_2	A: 7.53 (1H, *s*)<br>B: 7.06 (1H, *s*)	-
Phe	8	171.85	C	-	H-5, H-9, H10, H-5-NH
	9	54.71	CH	4.52 (1H, *m*)	H-10, H-9-NH
	10	38.03	CH_2	A: 2.98 (1H, *m*)<br>B: 2.74 (1H, *m*)	H-9, H-12
	11	138.06	C	-	H-9, H10, H-12, H-13
	12 + 12’	129.61	CH	7.21 (2H, *m*)	H-10, H-13, H-14
	13 + 13’	128.59	CH	7.22 (2H, *m*)	H-12, H-14
	14	126.81	CH	7.18 (1H, *m*)	H-12, H-13
	9-NH	-	NH	7.83 (1H, *d*, 8.2)	-
Gly (1)	15	169.27	C	-	H-9, H-16, H-9-NH, H-16-NH
	16	42.70	CH_2	A: 3.91 (1H, *m*)<br>B: 3.39 (1H, *m*)	H-16-NH
	16-NH	-	NH	8.13 (1H, *m*)	-
Glu	17	171.73	C	-	H-16, H-18, H-19, H-16-NH, H-18-NH
	18	53.34	CH	4.07 (1H, *m*)	H-19. H-20, H-18-NH
	19	26.63	CH_2	A: 1.91 (1H, *m*)<br>B: 1.81 (1H, *m*)	H-18, H-20
	20	30.63	CH_2	2.24 (2H, *m*)	H-18, H-19
	21	174.27	C	-	H-19, H-20
	18-NH	-	NH	8.39 (1H, *m*)	-
Val	22	172.29	C	-	H-18, H-23, H-24, H-18-NH
	23	58.61	CH	4.10 (1H, *m*)	H-24, H-25, H-26
	24	30.48	CH	1.98 (1H, *m*)	H-23, H-25, H-26
	25	19.59	CH_3	0.86 (3H, *d*, 6.8)	H-23, H-24, H-26
	26	19.06	CH_3	0.81 (3H, *d*, 6.7)	H-23, H-24, H-25
	23-NH	-	NH	7.62 (1H, *d*, 8.7)	-
Gly (2)	27	169.31	C	-	H-23, H-28, H-23-NH, H-28-NH
	28	43.05	CH_2	A: 3.91 (1H, *m*)<br>B: 3.62 (1H, *m*)	H-28-NH
	28-NH	-	NH	8.13 (1H. *m*)	-
Asn (2)	29	170.83	C	-	H-28, H-30, H-31, H-28-NH, H-30-NH

	30	50.73	CH	4.52 (1H, *m)*	H-31
	31	36.97	CH_2	A: 2.73 (1H, *m*)<br>B: 2.50 (1H, *m*)	H-30, H-32-NH_2
	32	172.25	C	-	H-30, H-31, H-32-NH_2
	30-NH	-	NH	9.09 (1H, *m*)	-
	32-NH_2	-	NH_2	A: 7.46 (1H, *s*)<br>B 6.94 (1H, *s*)	-
Cys (2)	33	167.82	C	-	H-30, H-34, H-35, H-30-NH
	34	52.50	CH	4.07 (1H, *m*)	H-35
	35	39.54	CH_2	A: 3.15 (1H, *m*)<br>B: 3.01 (1H, *m*)	-
	34-NH_2	-	NH_2	8.26 (2H, m)	-

Table S2 : ^{13}C NMR (100 MHz) and ^{1}H (400 MHz) NMR data for LCP_O in DMSO-d_6. ^{13}C values from carbon and HSQC spectra and ^{1}H values from proton and HSQC spectra.

Residue	Pos.	$^{13}C\delta$ (ppm)	C/N-Type	$^{1}H\delta$ (ppm) (Proton count, m, J (Hz))	HMBC Data ($^{13}C \rightarrow {}^{1}H$)
Cys (1)	1	171.79	C	-	H-2, H-3, H-2-NH, H-1-NH_2
	2	51.85	CH	4.44 (1H, *m*)	H-3, H-2-NH, H-1-NH_2
	3	40.66	CH_2	A: 3.12 (1H, *m*)<br>B: 3.00 (1H, *m*)	H-2, H-2-NH
	2-NH	-	NH	8.15 (1H, *m*)	-
	1-NH_2	-	NH_2	A: 7.48 (1H, *s*)<br>B: 7.32 (1H, *s*)	-
Asn (1)	4	170.81	C	-	H-2, H-5, H-6, H-2-NH, H-5-NH
	5	49.84	CH	4.57 (1H, *m*)	H-6, H-5-NH
	6	36.52	CH_2	A: 2.65 (1H, *m*)<br>B: 2.49 (1H, *m*)	H-5, H-7-NH_2
	7	171.51	C	-	H-6, H-5, H-7-NH_2
	5-NH	-	NH	8.42 (1H, *d*, 7.6)	-
	7-NH_2	-	NH_2	A: 7.52 (1H, *s*)<br>B: 7.06 (1H, *s*)	-
Phe	8	171.37	C	-	H-5, H-9, H-10, H-5-NH
	9	54.17	CH	4.52 (1H, *m*)	H-10, H-9-NH
	10	37.59	CH_2	A: 2.97 (1H, *m*)<br>B: 2.74 (1H, *m*)	H-9, H-12, H-9-NH
	11	137.63	C	-	H-9, H-10, H-12, H-13
	12 + 12’	129.17	CH	7.22 (2H, *m*)	H-10, H-13, H-14
	13 + 13’	128.10	CH	7.24 (2H, *m*)	H-12, H-14
	14	126.34	CH	7.18 (1H, *m*)	H-12, H-13
	9-NH	-	NH	7.81 (1H, *d*, 8.6)	-
Gly (1)	15	168.85	C	-	H-9, H-16, H-9-NH, H-16-NH
	16	42.24	CH_2	A: 3.91 (1H, *m*)<br>B: 3.39 (1H, *m*)	H-16-NH
	16-NH	-	NH	8.17 (1H, *m*)	-
Ala	17	172.43	C	-	H-16, H-18, H-19, H-16-NH, H-18-NH
	18	49.03	CH	4.12 (1H, *m*)	H-19, H-18-NH
	19	17.12	CH_3	1.23 (3H, *d*, 7.0)	H-18, H-18-NH
	18-NH	-	NH	8.38 (1H, *d*, 5.6)	-
Val	20	171.87	C	-	H-18, H-21, H-22, H-18-NH
	21	58.03	CH	4.09 (1H, *m*)	H-22, H-23, H-24, H-21-NH
	22	30.00	CH	1.99 (1H, *m)*	H-21, H-23, H-24
	23	19.12	CH_3	0.86 (3H, *d*, 6.7)	H-21, H-22, H-24
	24	18.62	CH_3	0.81 (3H, *d*, 6.9)	H-21, H-22, H-23
	21-NH	-	NH	7.60 (1H, *d*, 8.6)	-
Gly (2)	25	168.79	C	-	H-21, H-26, H-21-NH, H-26-NH
	26	42.60	CH_2	A: 3.91 (1H, *m*)<br>B: 3.59 (1H, *m*)	H-26-NH
	26-NH	-	NH	8.14 (1H, *m*)	-
Asn (2)	27	170.38	C	-	H-26, H-28, H-29, H-26-NH, H-28-NH
	28	50.28	CH	4.52 (1H, *m*)	H-29, H-28-NH
	29	36.15	CH_2	A: 2.71 (1H, *m*)<br>B: 2.49 (1H, *m*)	H-28, H-30-NH_2

	30	171.13	C	-	H-28, H-29, H-30-NH_2
	28-NH	-	NH	9.09 (1H, *d*, 7.1)	-
	30-NH_2	-	NH_2	A: 7.46 (1H, *s*)	-
				B: 6.92 (1H, *s*)	
Cys (2)	31	167.37	C	-	H-28, H-33, H-28-NH, H-32-NH_2
	32	51.99	CH	4.09 (1H, *m*)	H-33
	33	38.96	CH_2	A: 3.16 (1H, *m*)	-
				B: 3.02 (1H, *m*)	
	32-NH_2	-	NH_2	8.26 (2H, *m*)	-

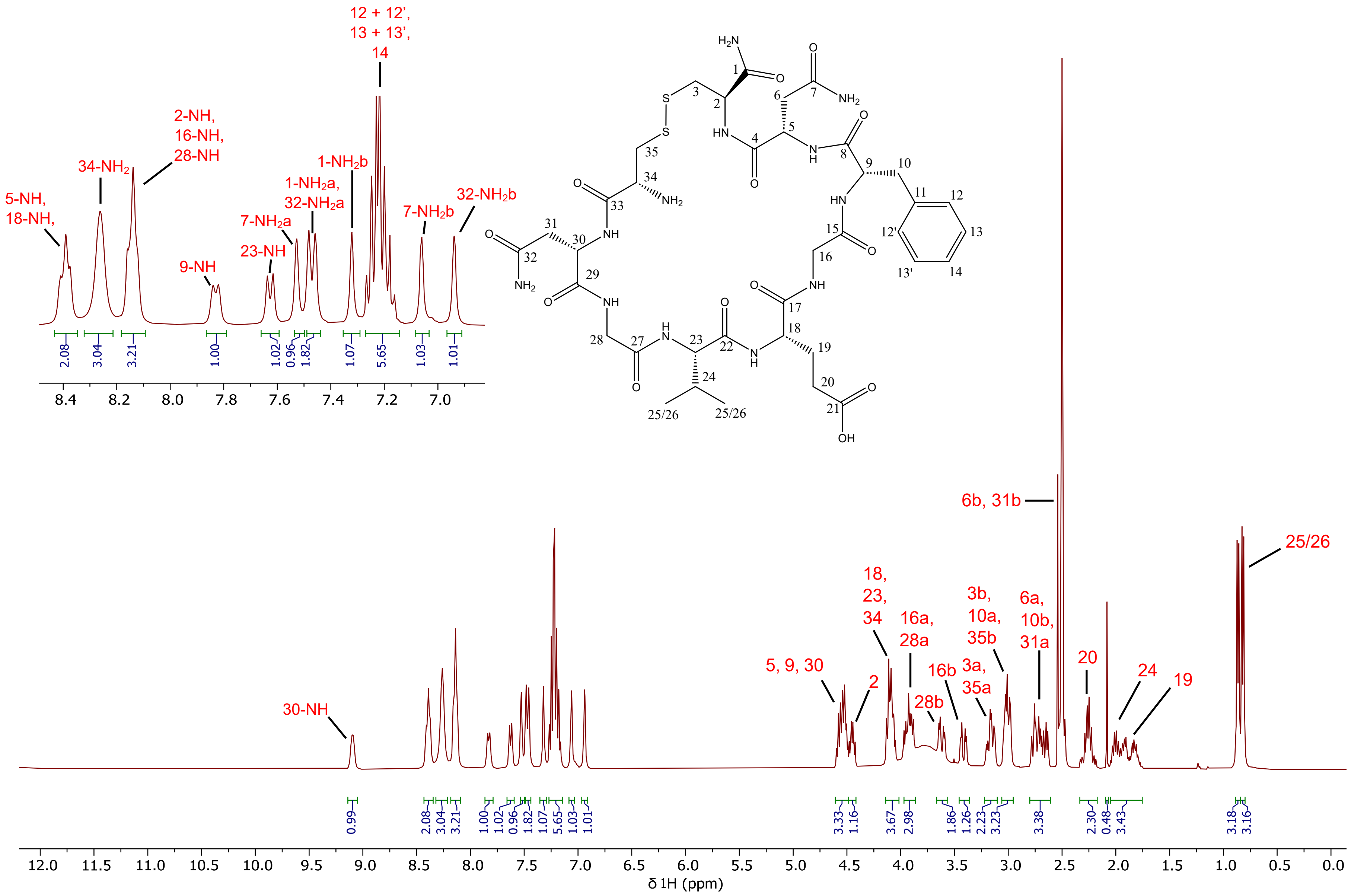

Figure S3: ^{1}H NMR (400 MHz) spectrum of LCP$_W$ in DMSO-d_6.

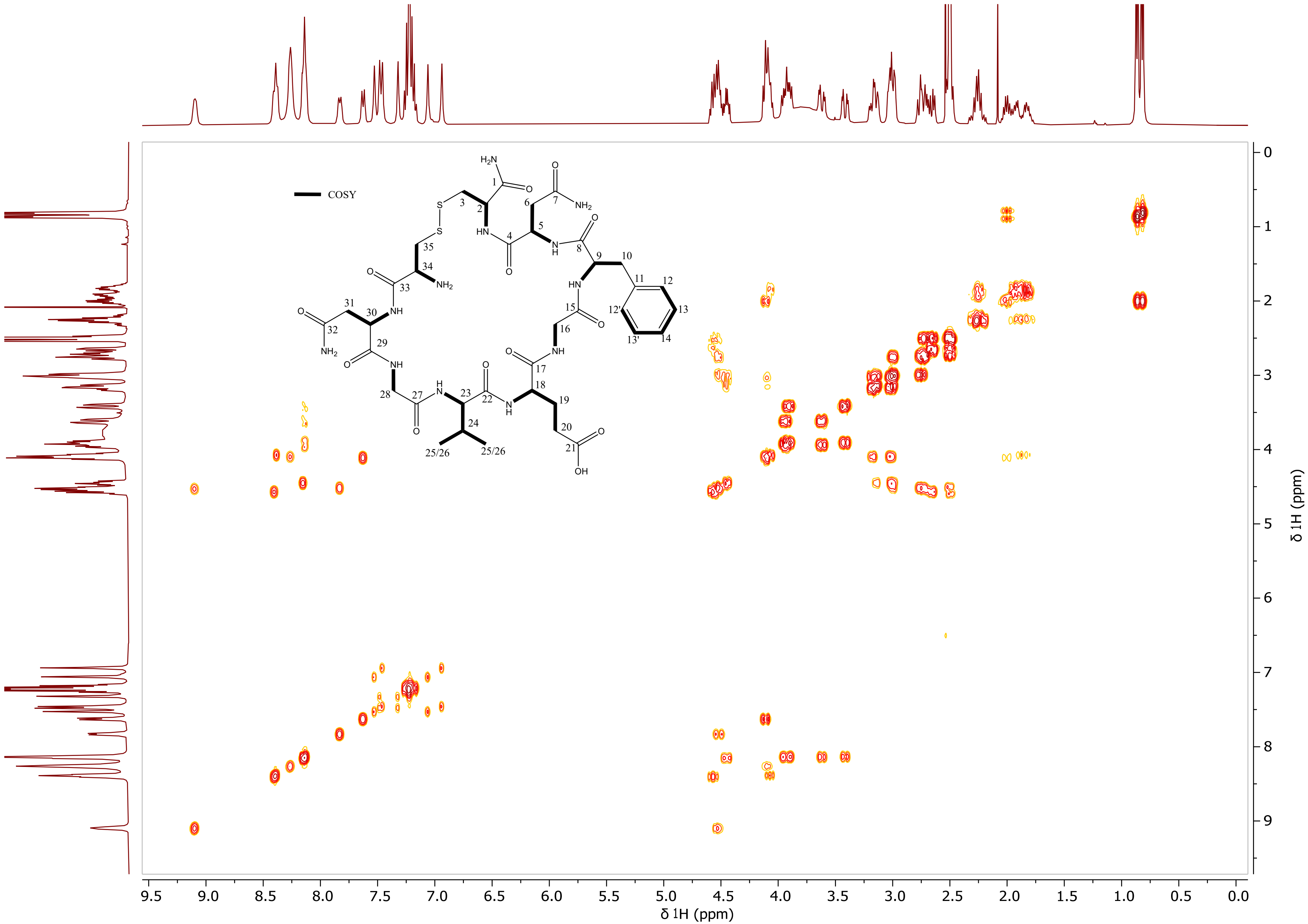

Figure S4: ^{1}H-^{1}H COSY NMR (400 MHz) full spectrum of LCP$_W$ in DMSO-d_6

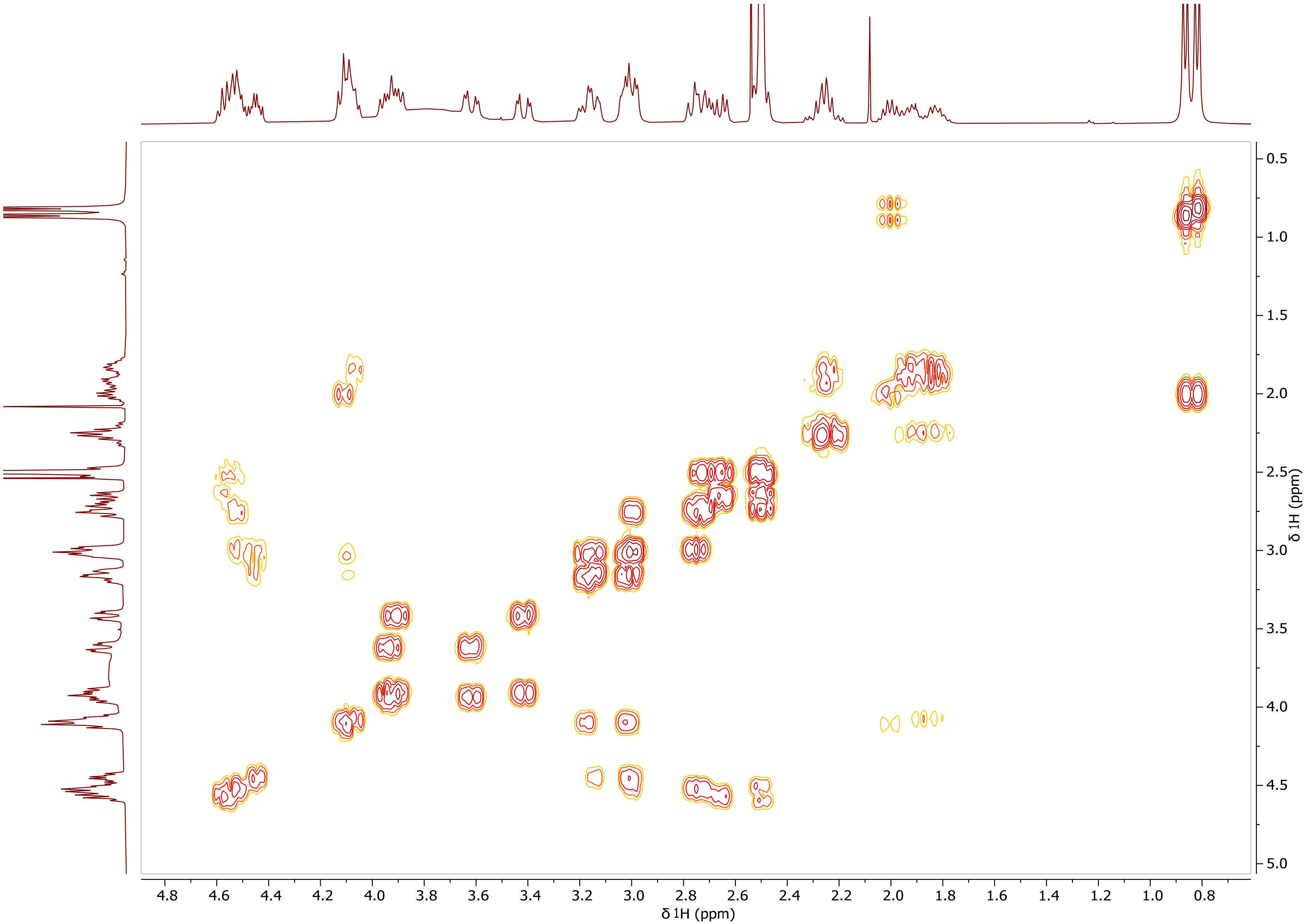

Figure S5: ^{1}H-^{1}H COSY NMR (400 MHz) spectrum of LCP_W in DMSO-d_6

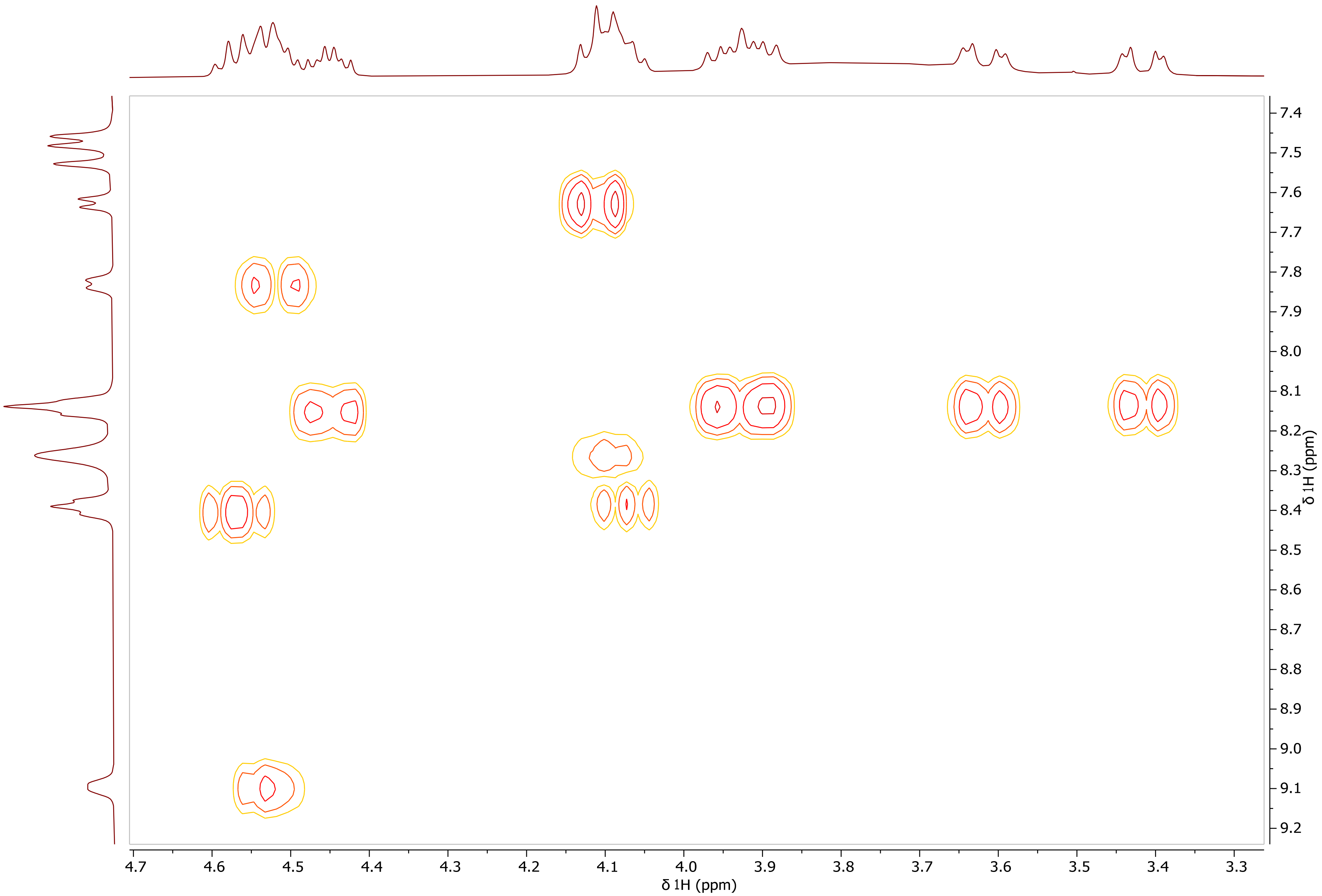

Figure S6: ^{1}H-^{1}H COSY NMR (400 MHz) spectrum of LCP_W in DMSO-d_6

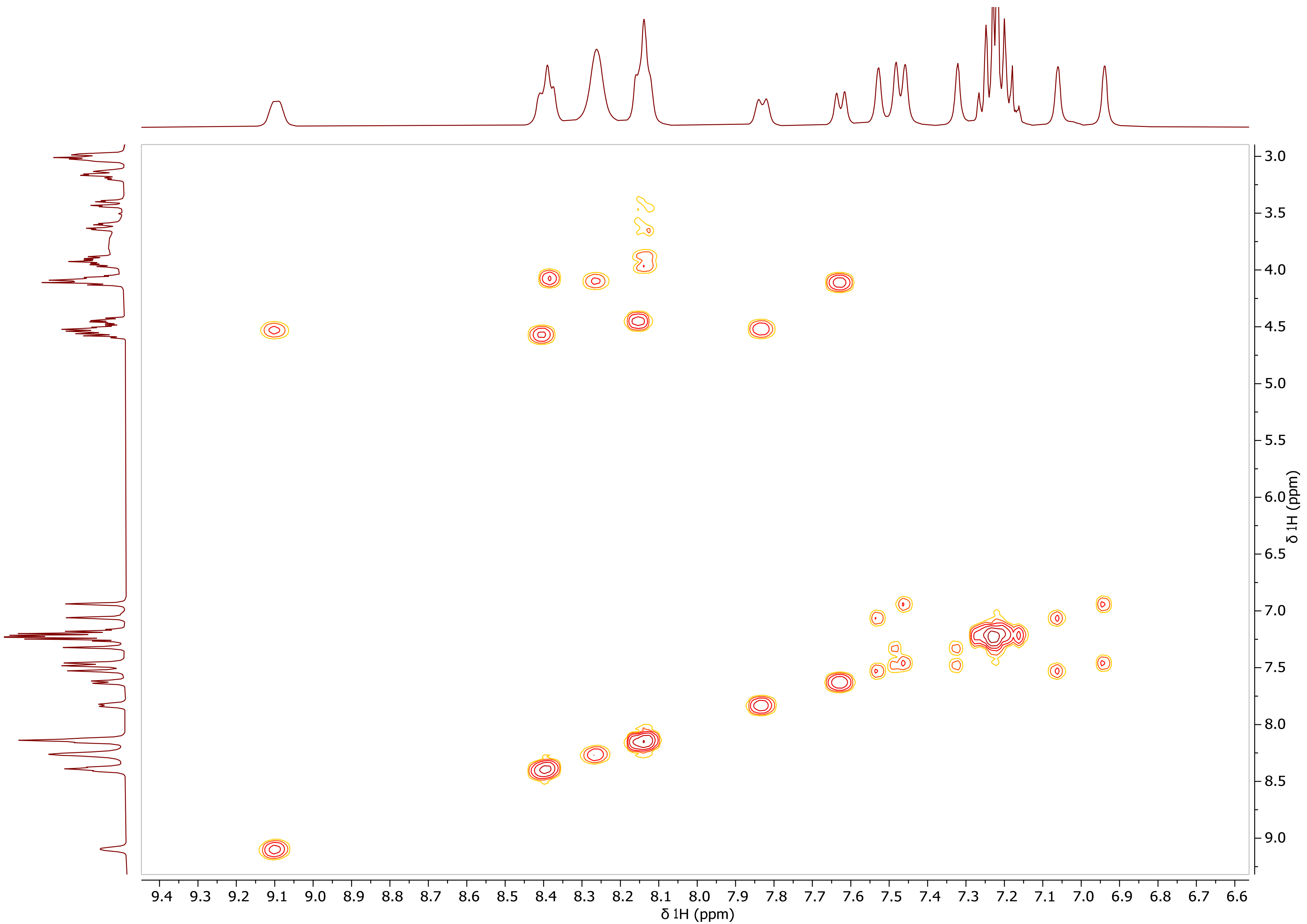

Figure S7: ^{1}H-^{1}H COSY NMR (400 MHz) spectrum of LCP_W in DMSO-d_6

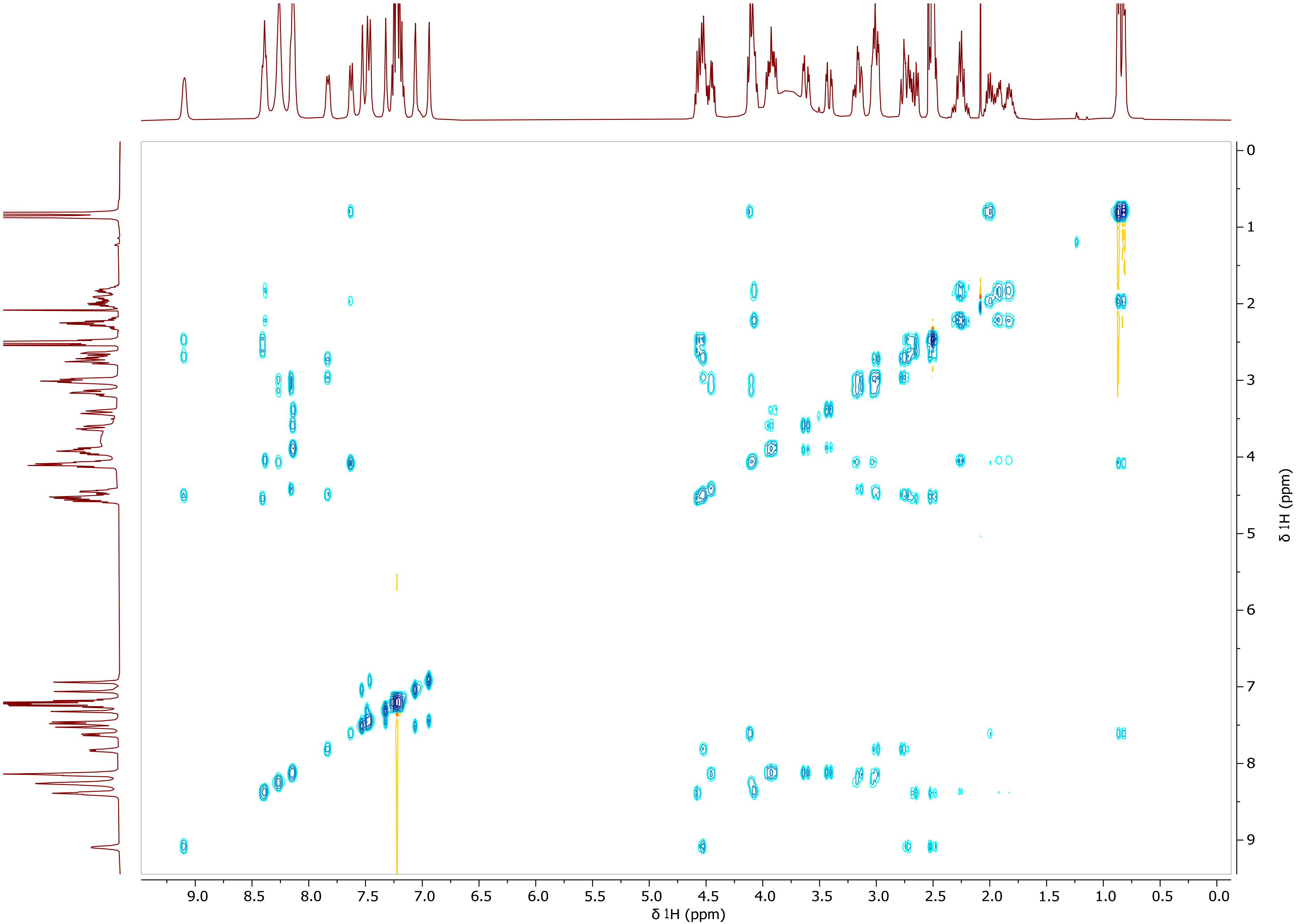

Figure S8: ^{1}H-^{1}H TOCSY NMR (400 MHz) spectrum of LCP_W in DMSO-d_6

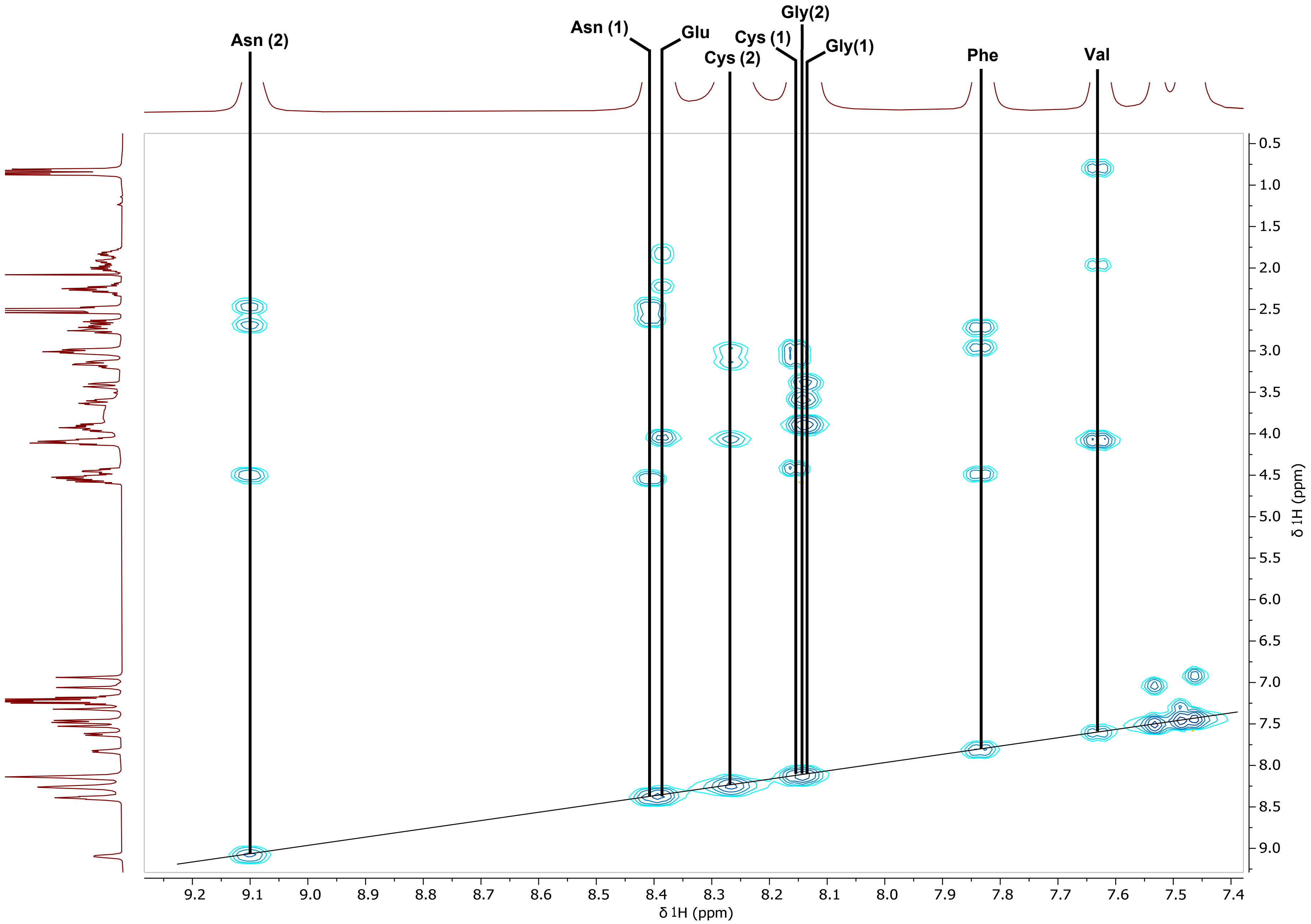

Figure S9: ^{1}H-^{1}H TOCSY NMR (400 MHz) spectrum of LCP_W in DMSO-d_6

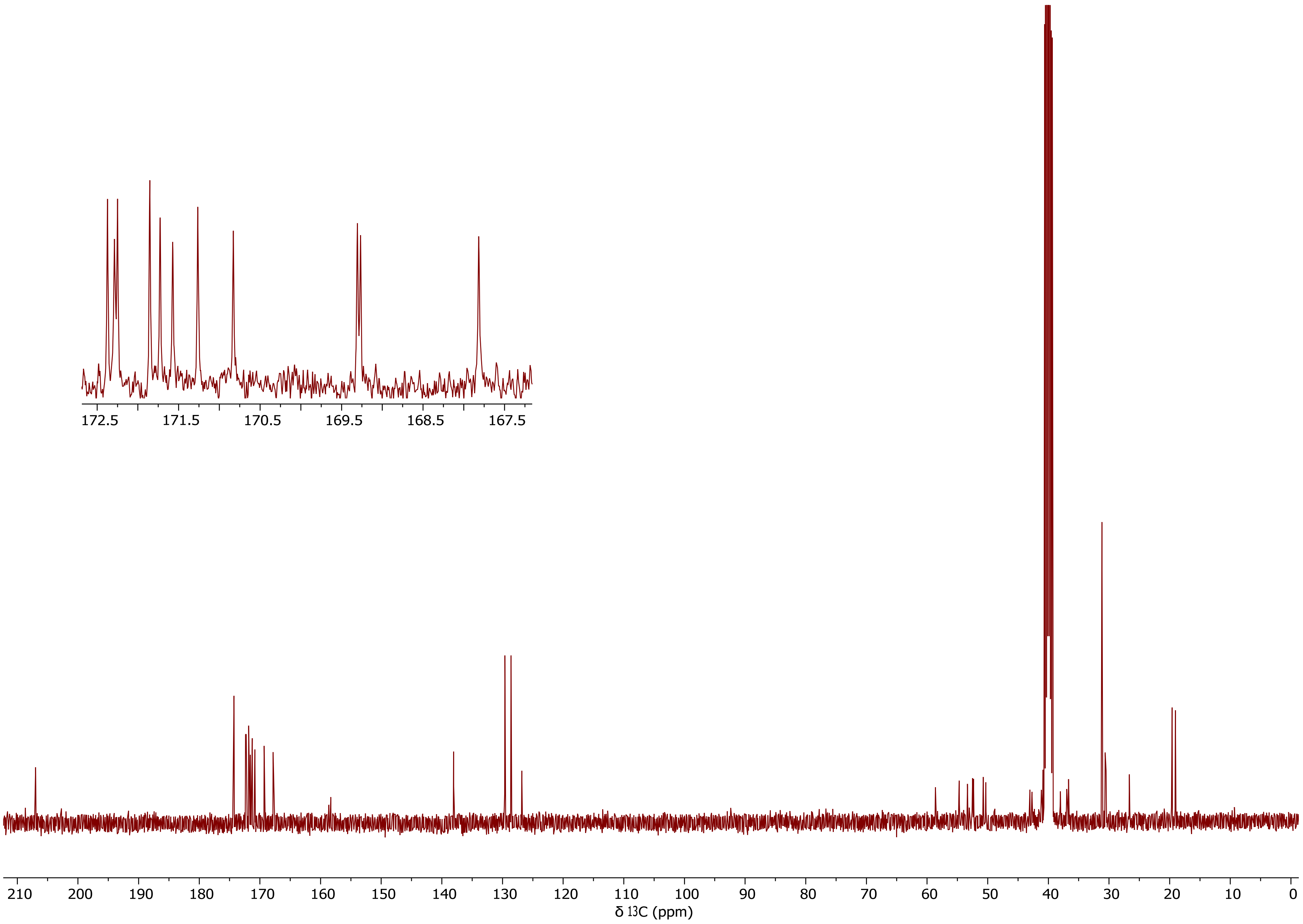

Figure S10: ^{13}C NMR (100 MHz) spectrum of LCP_W in DMSO-d_6

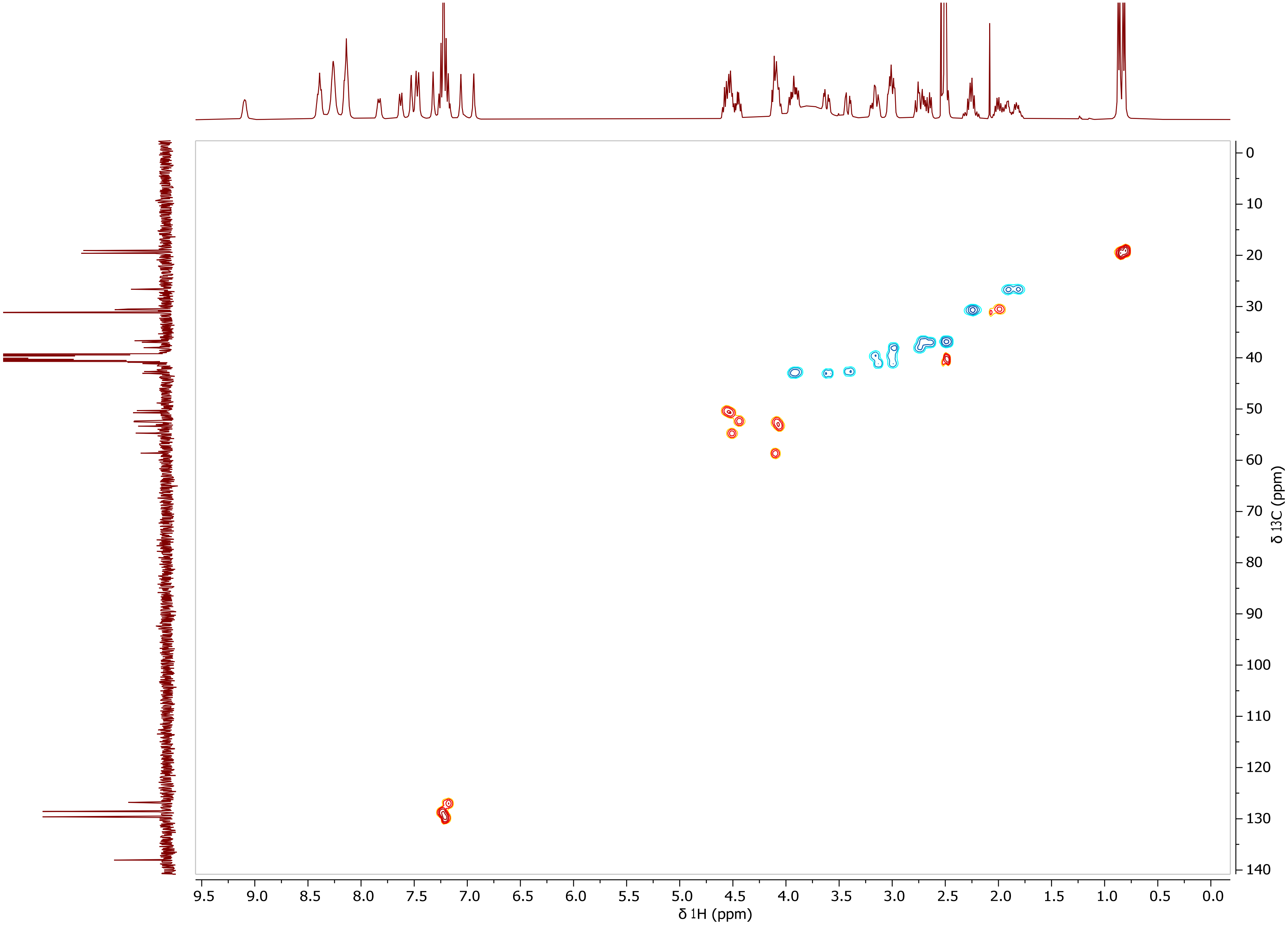

Figure S11: HSQC NMR (400 MHz) full spectrum of LCP_W in DMSO-d_6

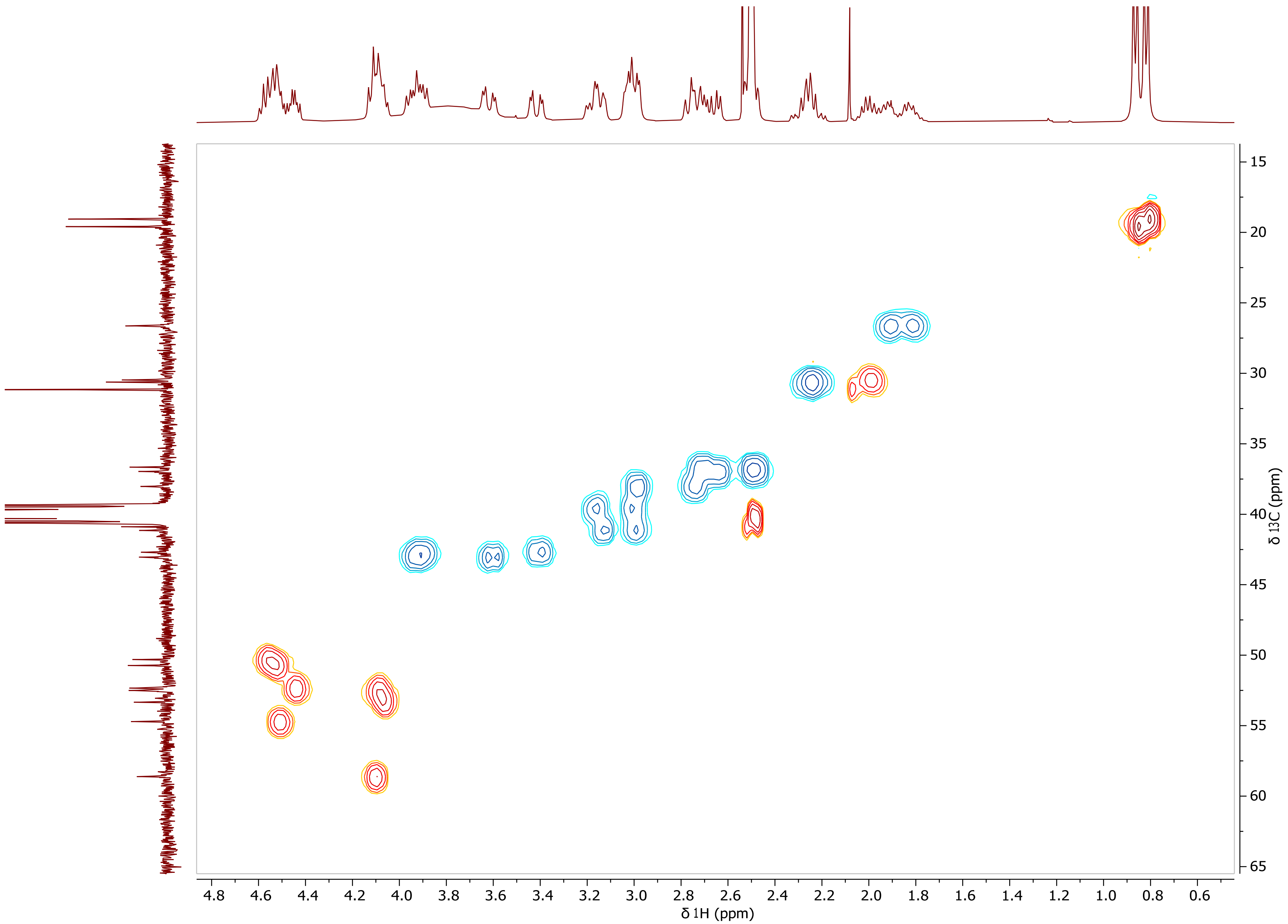

Figure S12: HSQC NMR (400 MHz) spectrum of LCP_W in DMSO-d_6

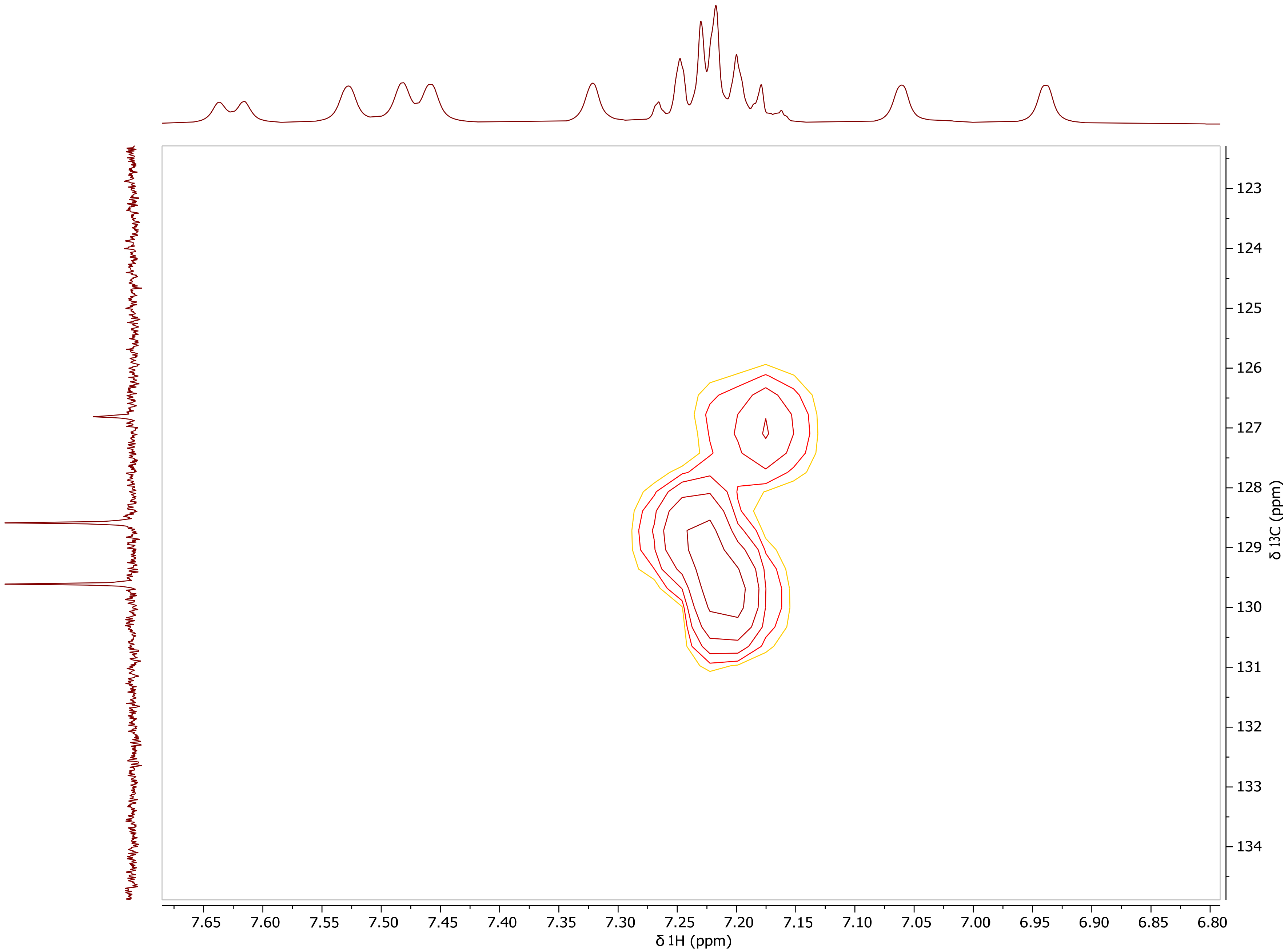

Figure S13: HSQC NMR (400 MHz) spectrum of LCP_W in DMSO-d_6

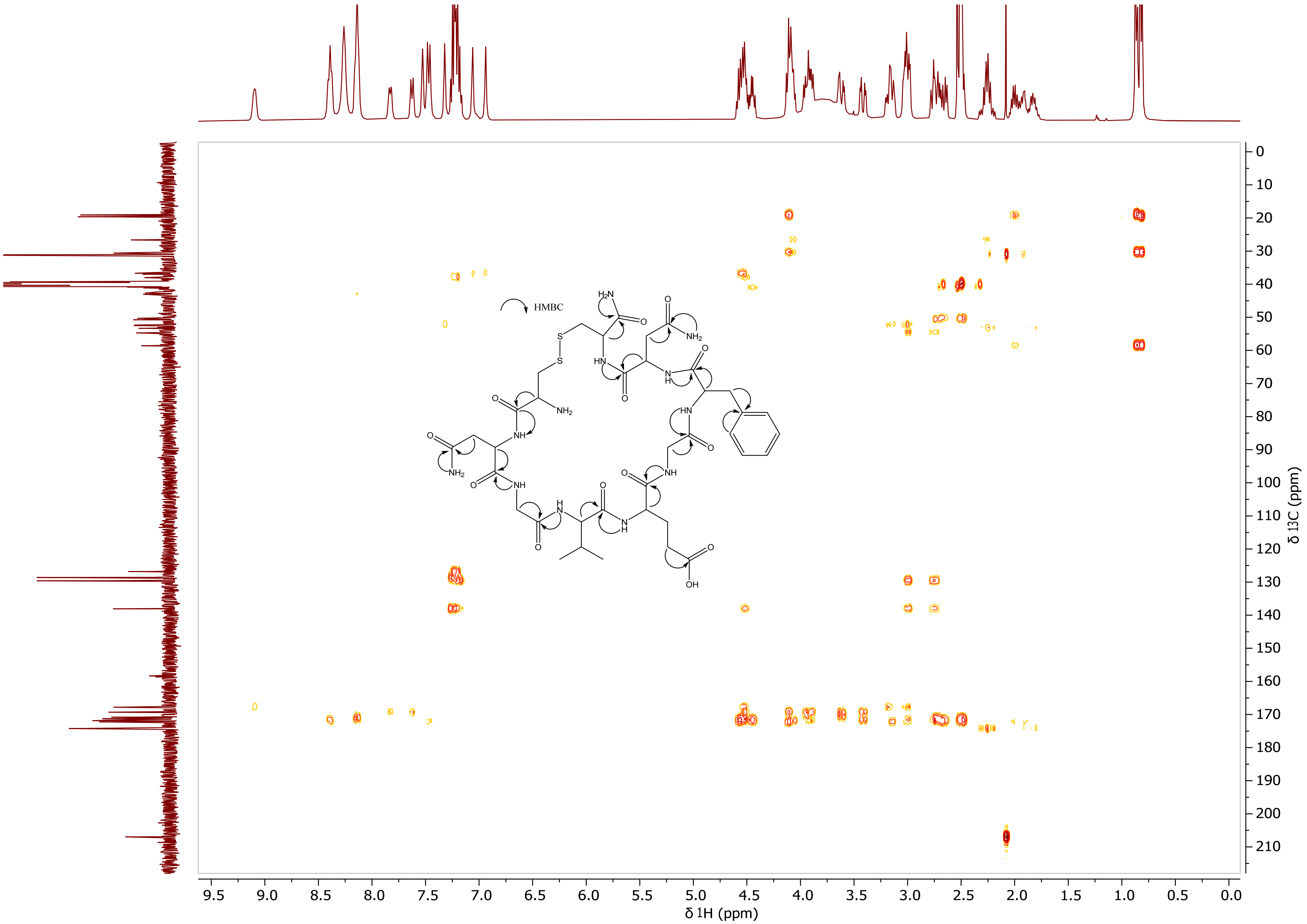

Figure S14: HMBC NMR (400 MHz) full spectrum of LCP_W in DMSO-d_6

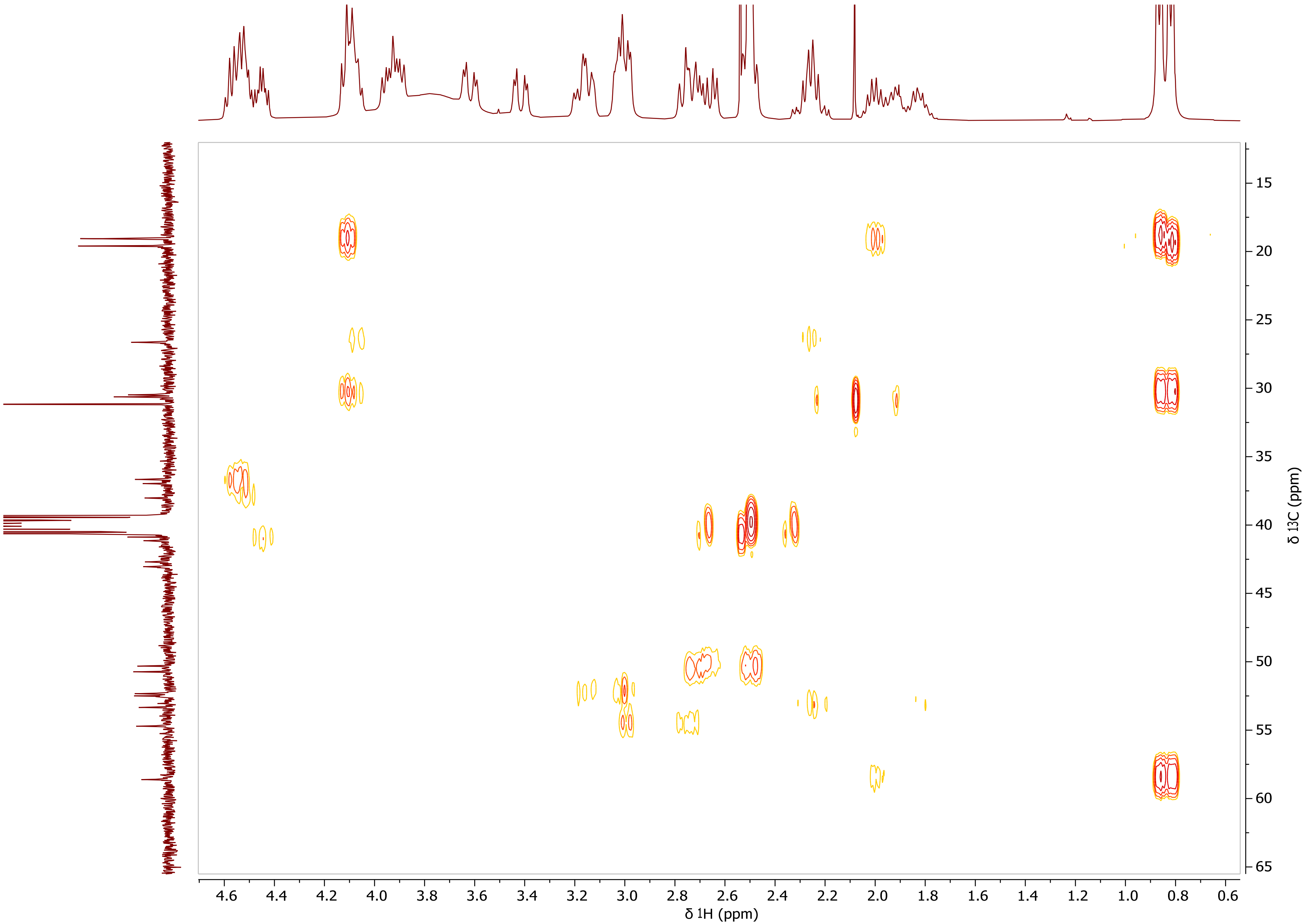

Figure S15: HMBC NMR (400 MHz) spectrum of LCP_W in DMSO-d_6

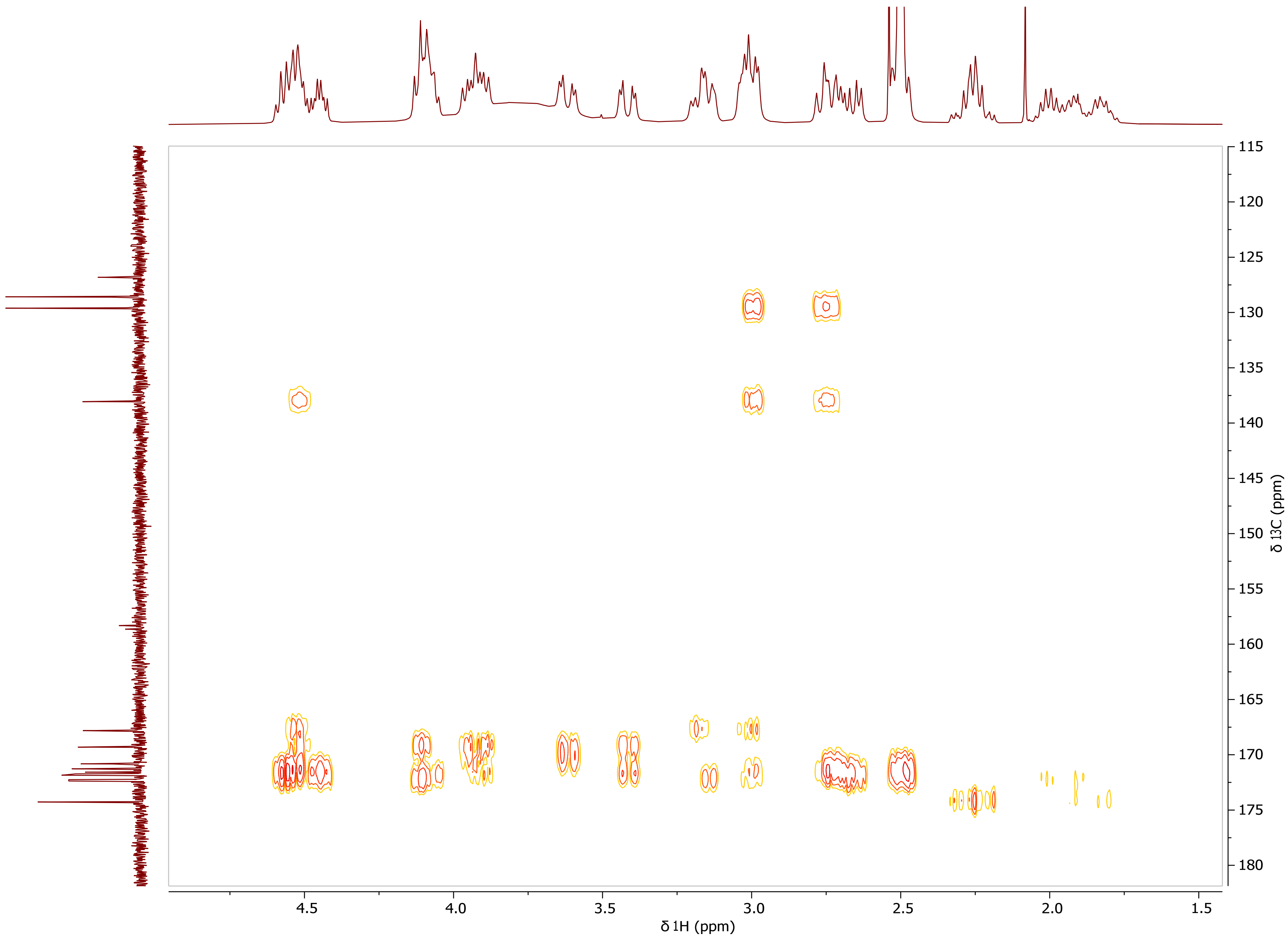

Figure S16: HMBC NMR (400 MHz) spectrum of LCP_W in DMSO-d_6

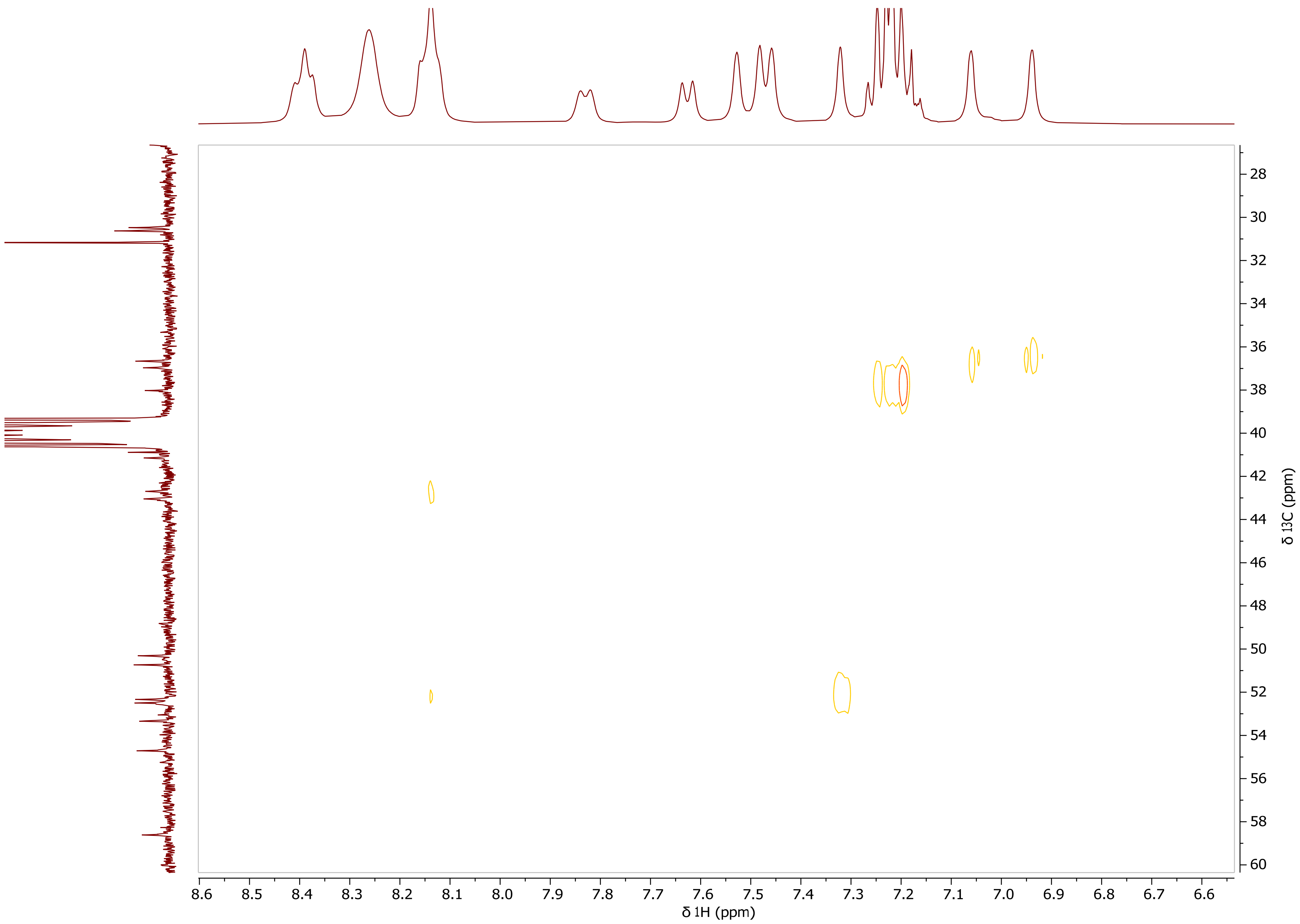

Figure S17: HMBC NMR (400 MHz) spectrum of LCP_W in DMSO-d_6

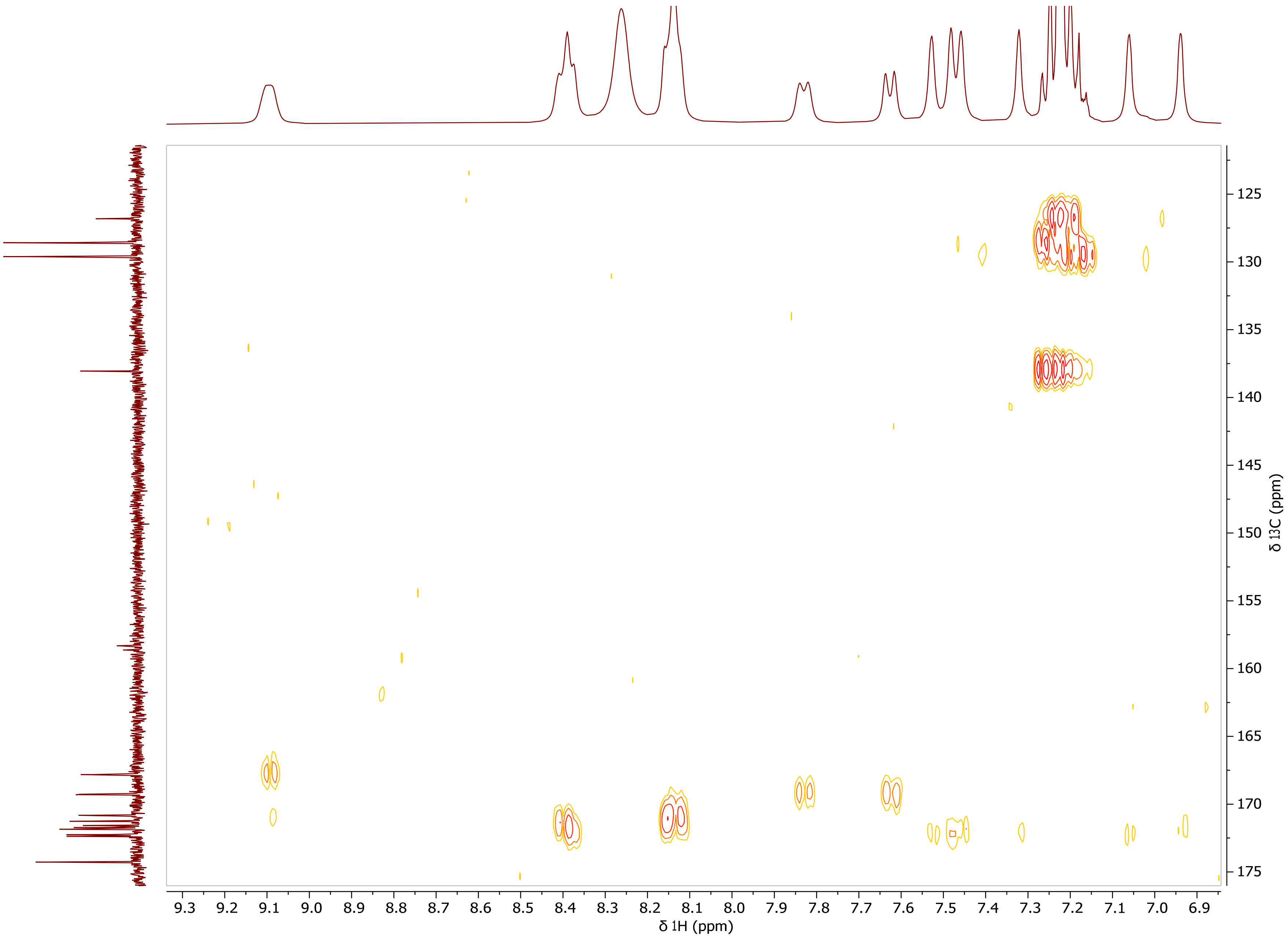

Figure S18: HMBC NMR (400 MHz) spectrum of LCP_W in DMSO-d_6

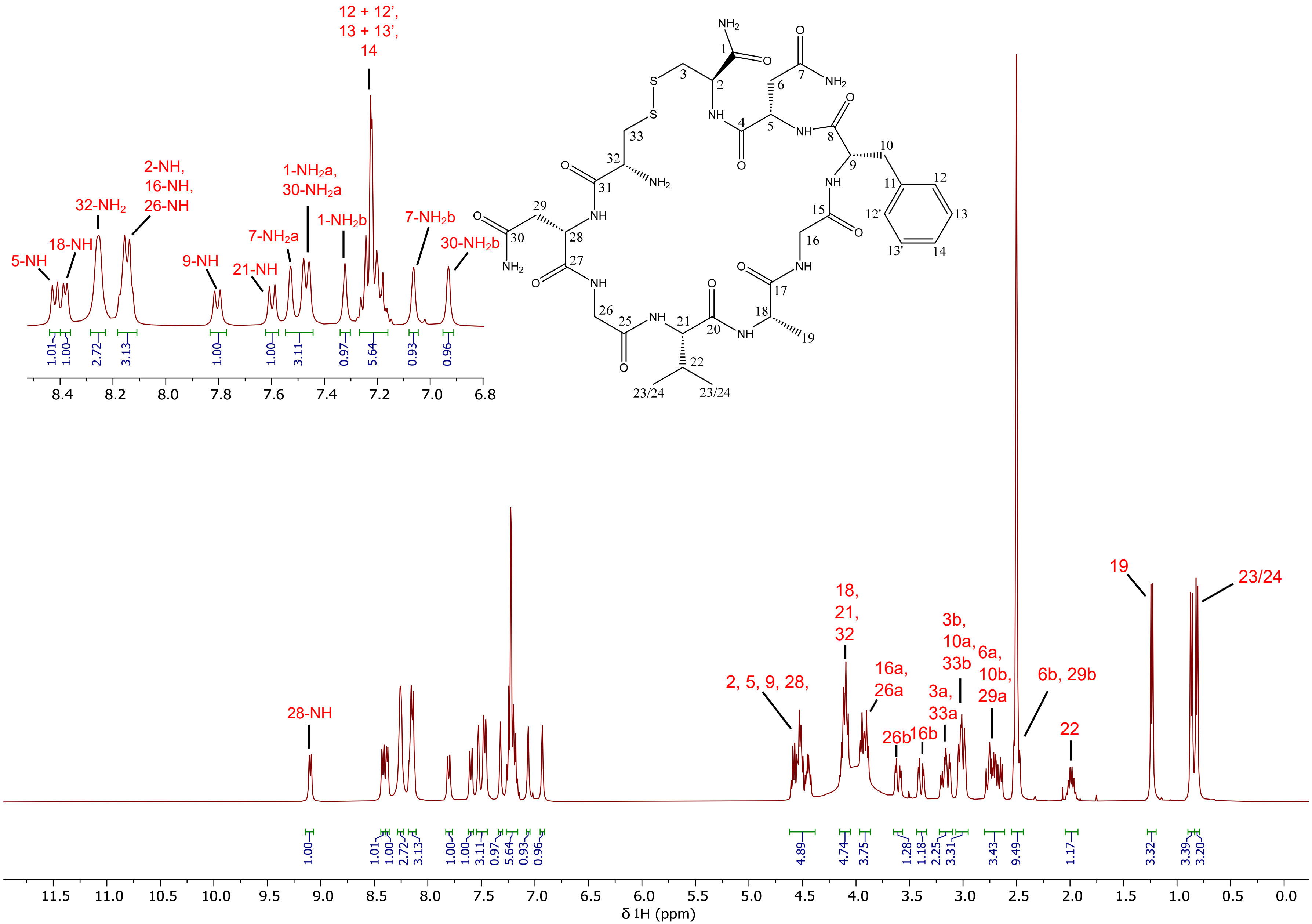

Figure S19: ^{1}H NMR (400 MHz) spectrum of LCP$_O$ in DMSO-d_6.

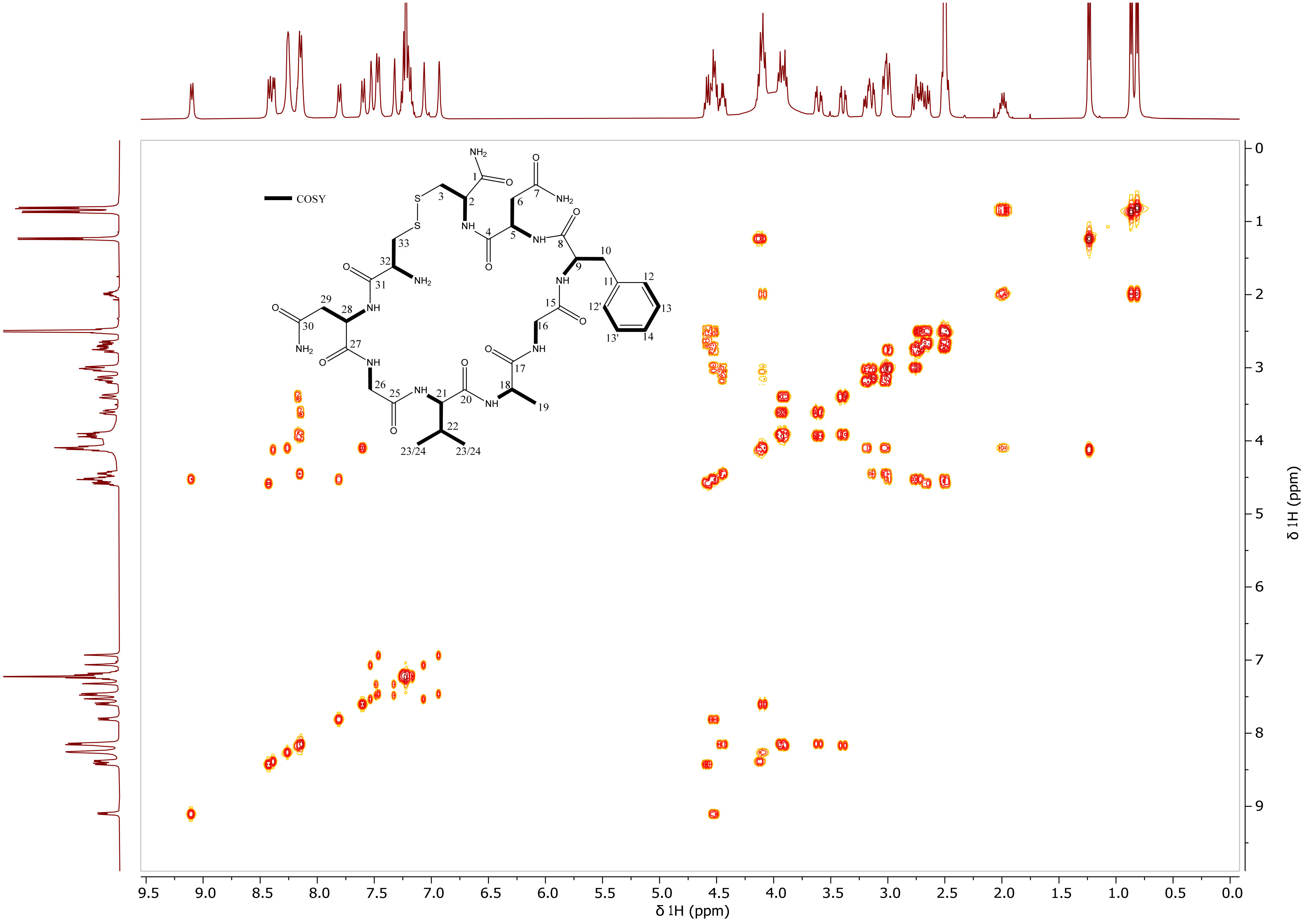

Figure S20: ^{1}H-^{1}H COSY NMR (400 MHz) full spectrum of LCP_O in DMSO-d_6

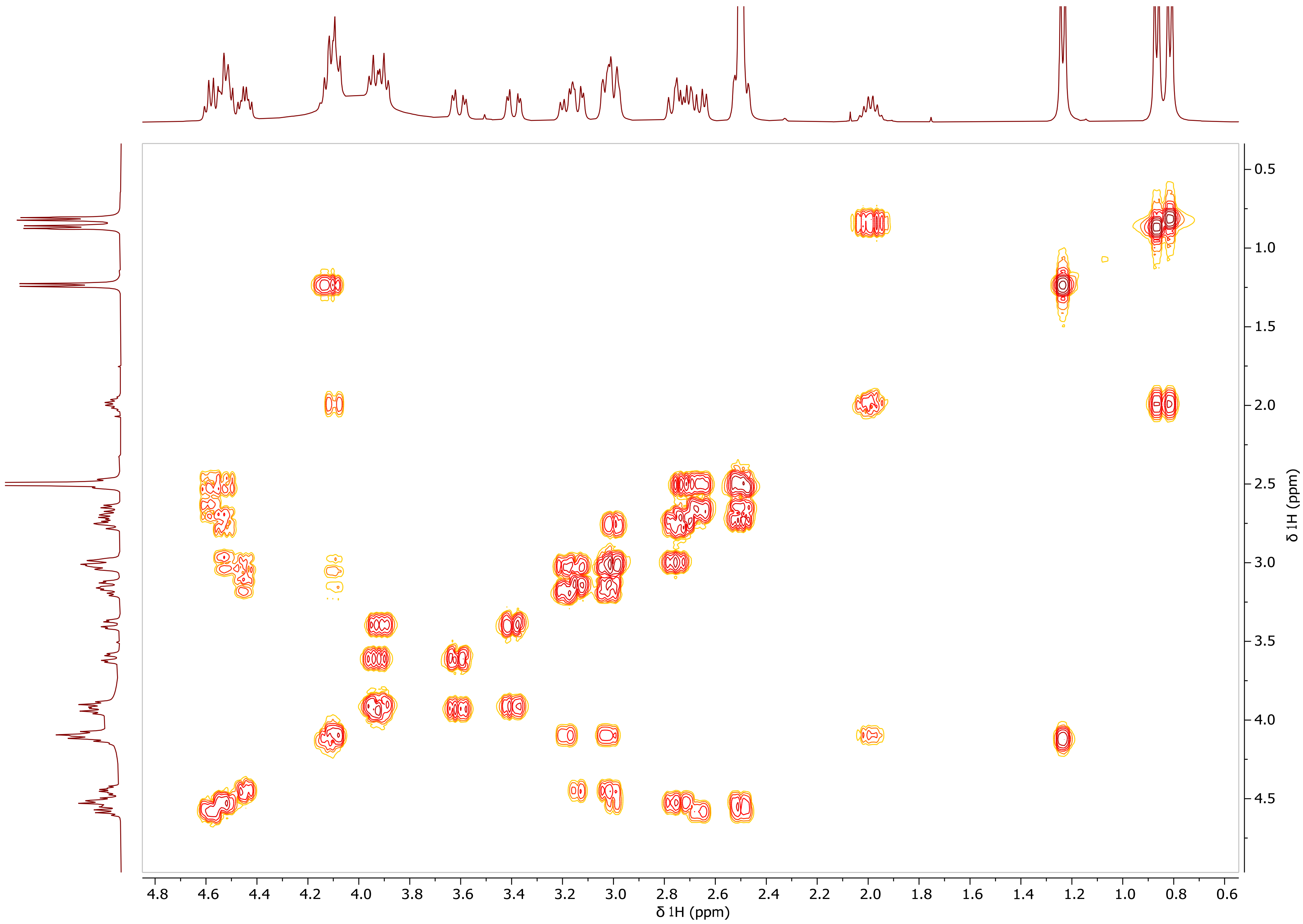

Figure S21: ^1H-^1H COSY NMR (400 MHz) spectrum of LCP_O in DMSO-d_6

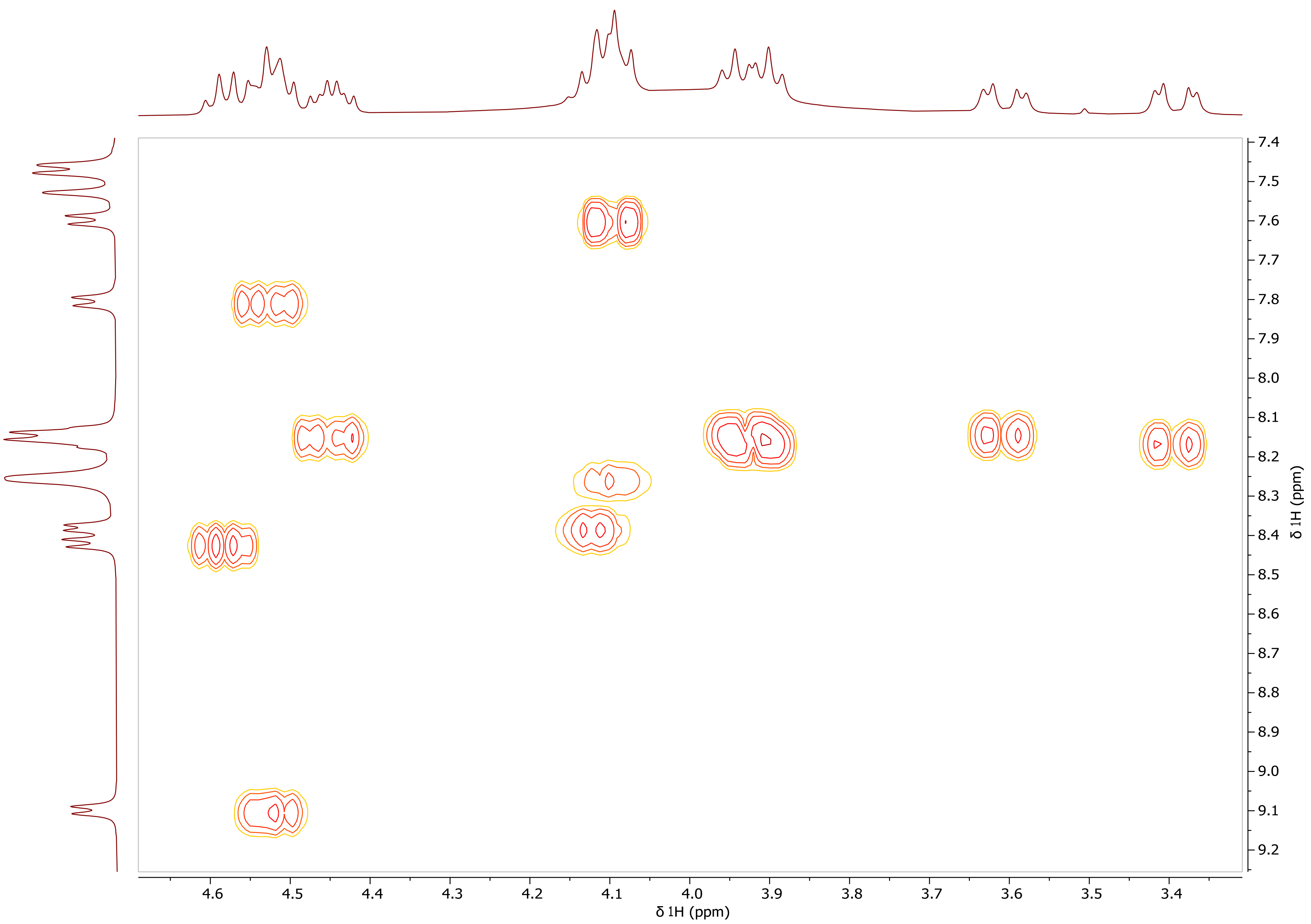

Figure S22: ^1H-^1H COSY NMR (400 MHz) spectrum of LCP_O in DMSO-d_6

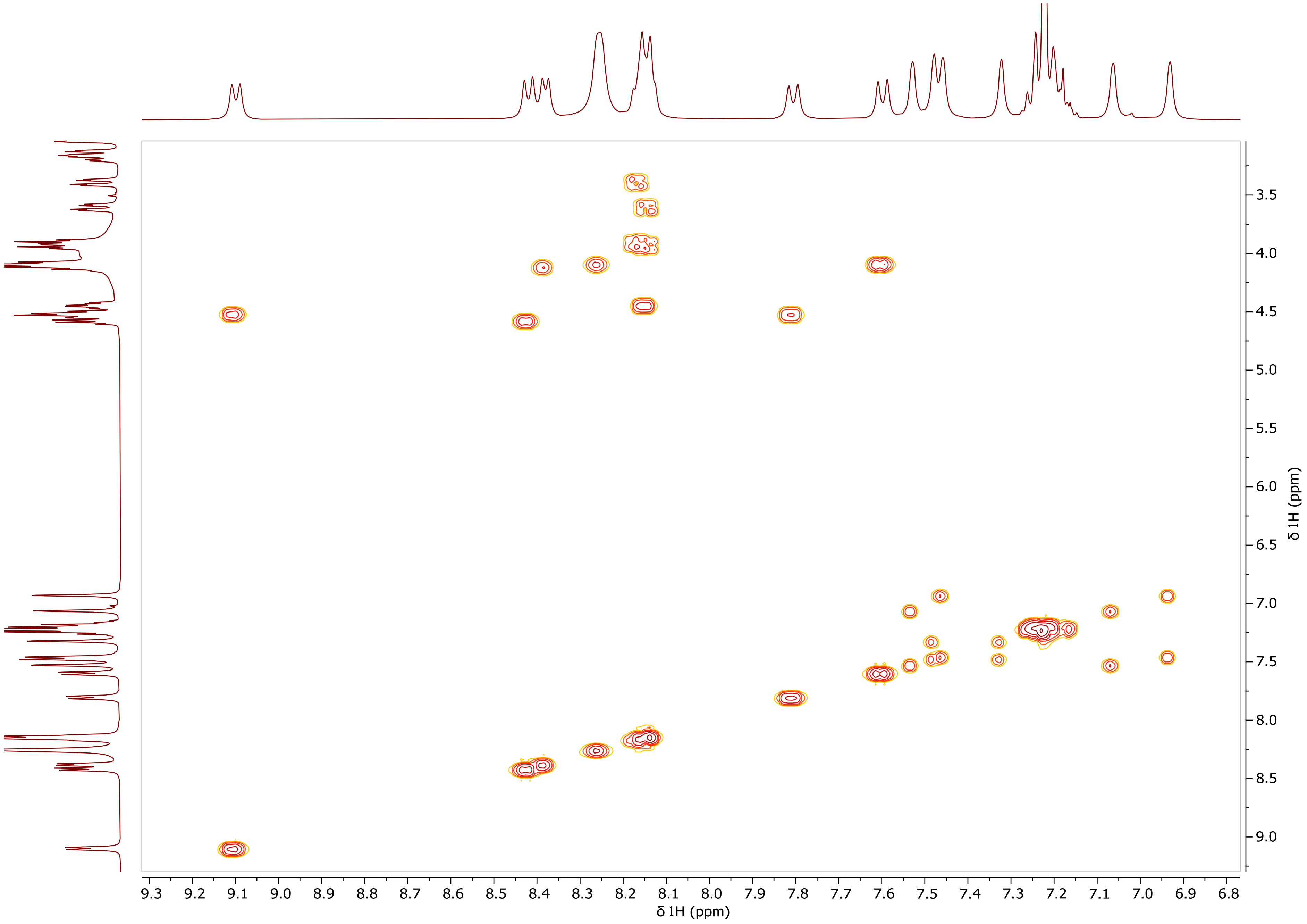

Figure S23: ^1H-^1H COSY NMR (400 MHz) spectrum of LCP_O in DMSO-d_6

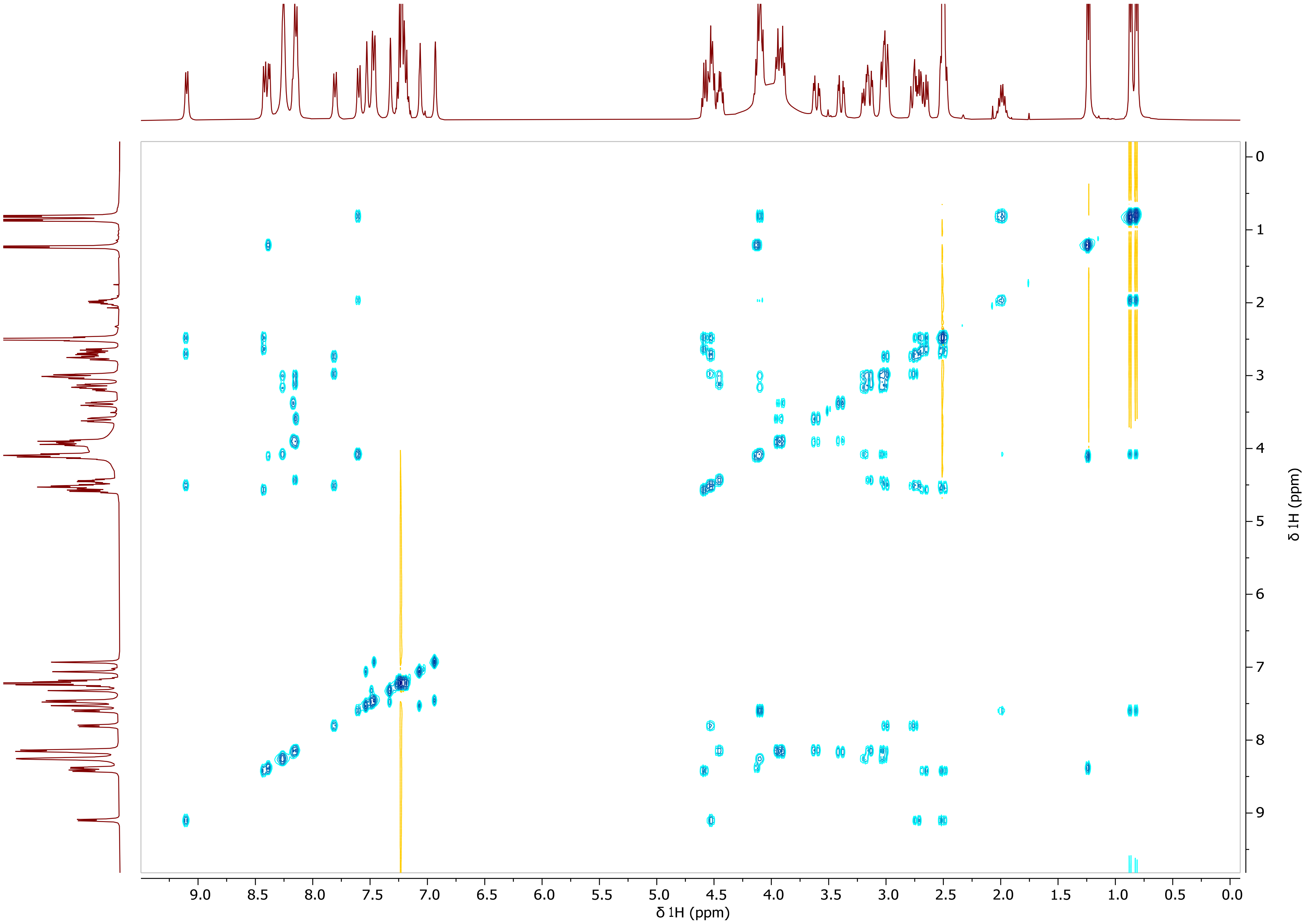

Figure S24: ^{1}H-^{1}H TOCSY NMR (400 MHz) spectrum of LCP_O in DMSO-d_6

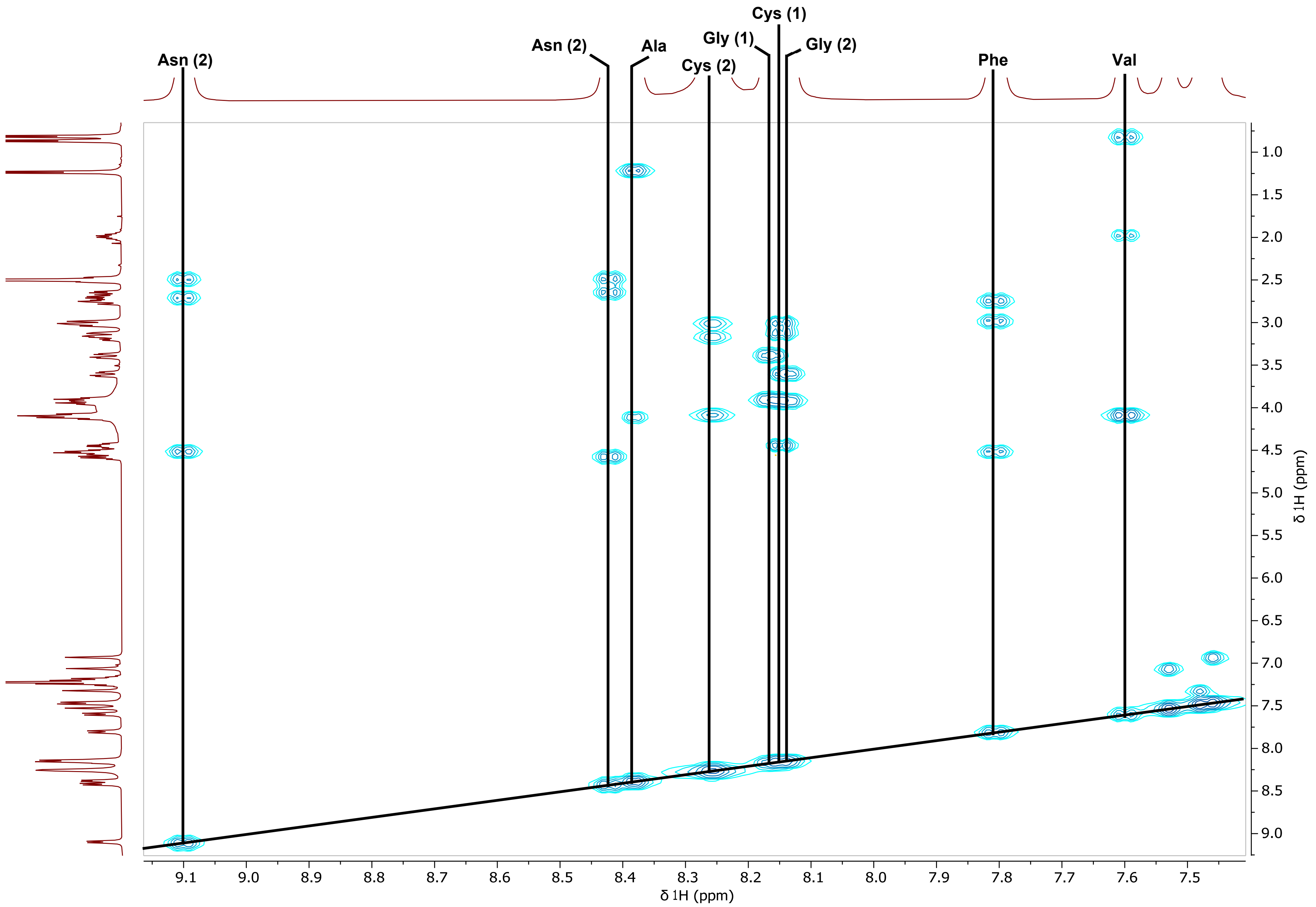

Figure S25: ^1H-^1H TOCSY NMR (400 MHz) spectrum of LCP_O in DMSO-d_6

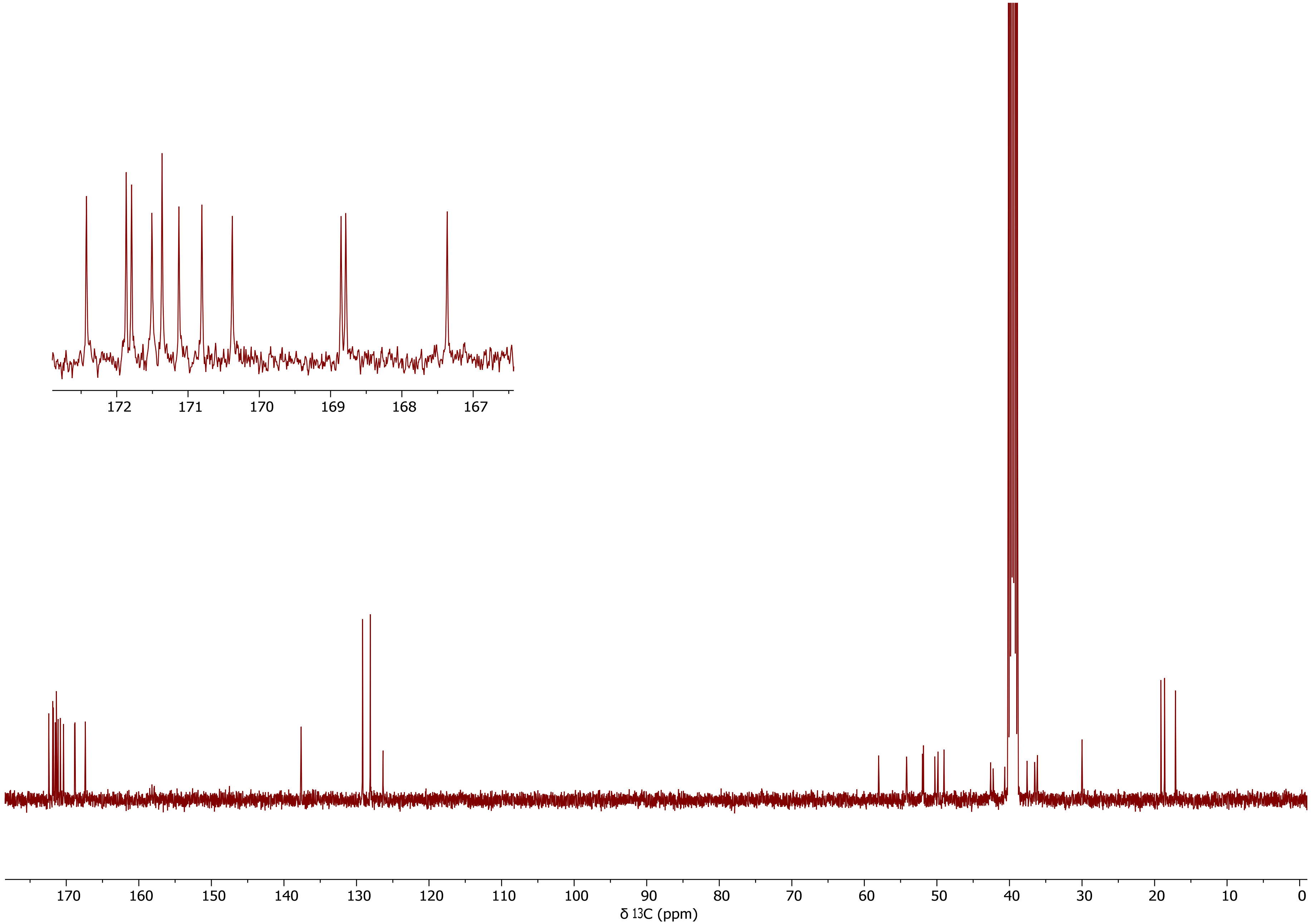

Figure S26: ^{13}C NMR (100 MHz) spectrum of LCP_O in DMSO-d_6

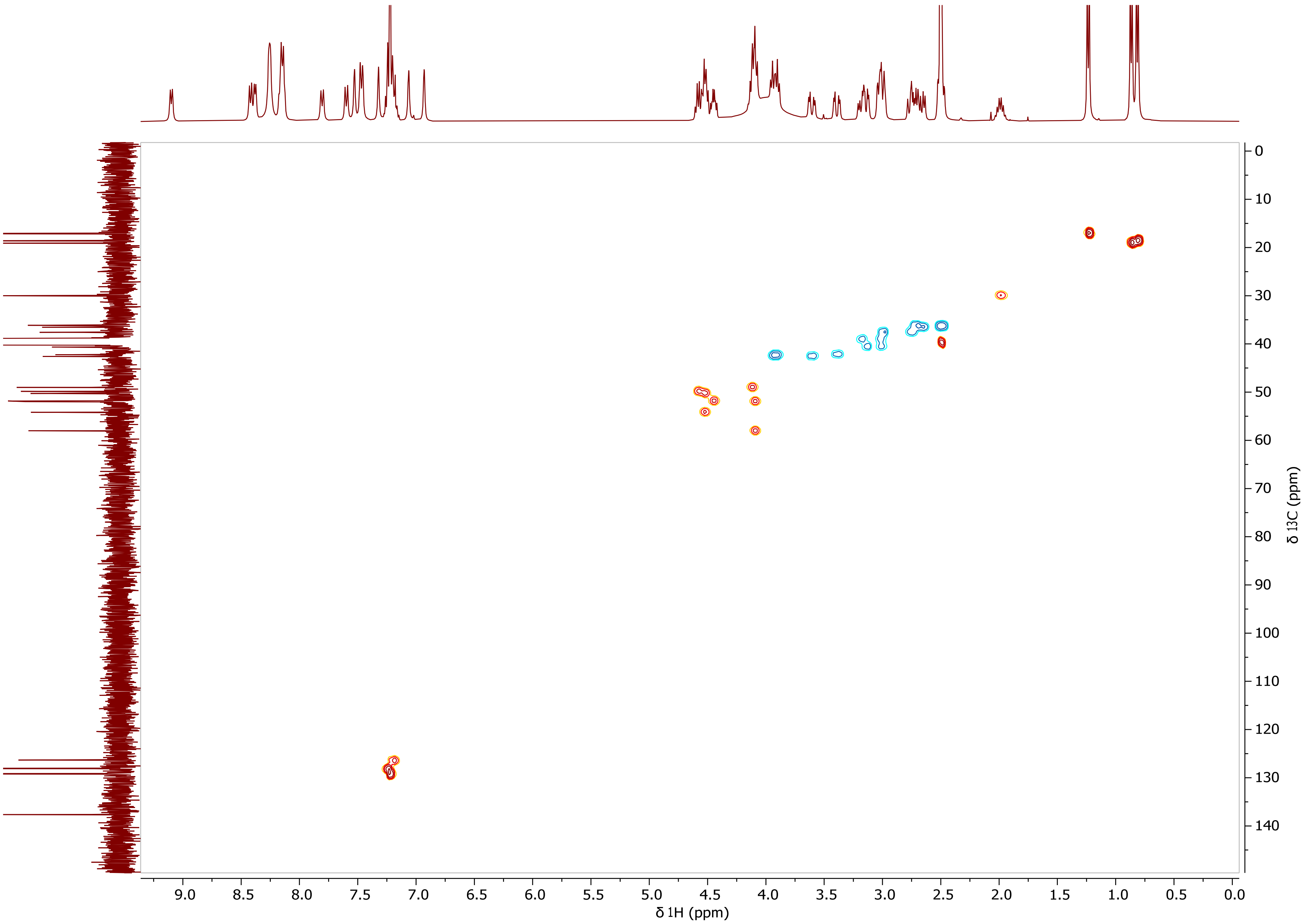

Figure S27: HSQC NMR (400 MHz) full spectrum of LCP_O in DMSO-d_6

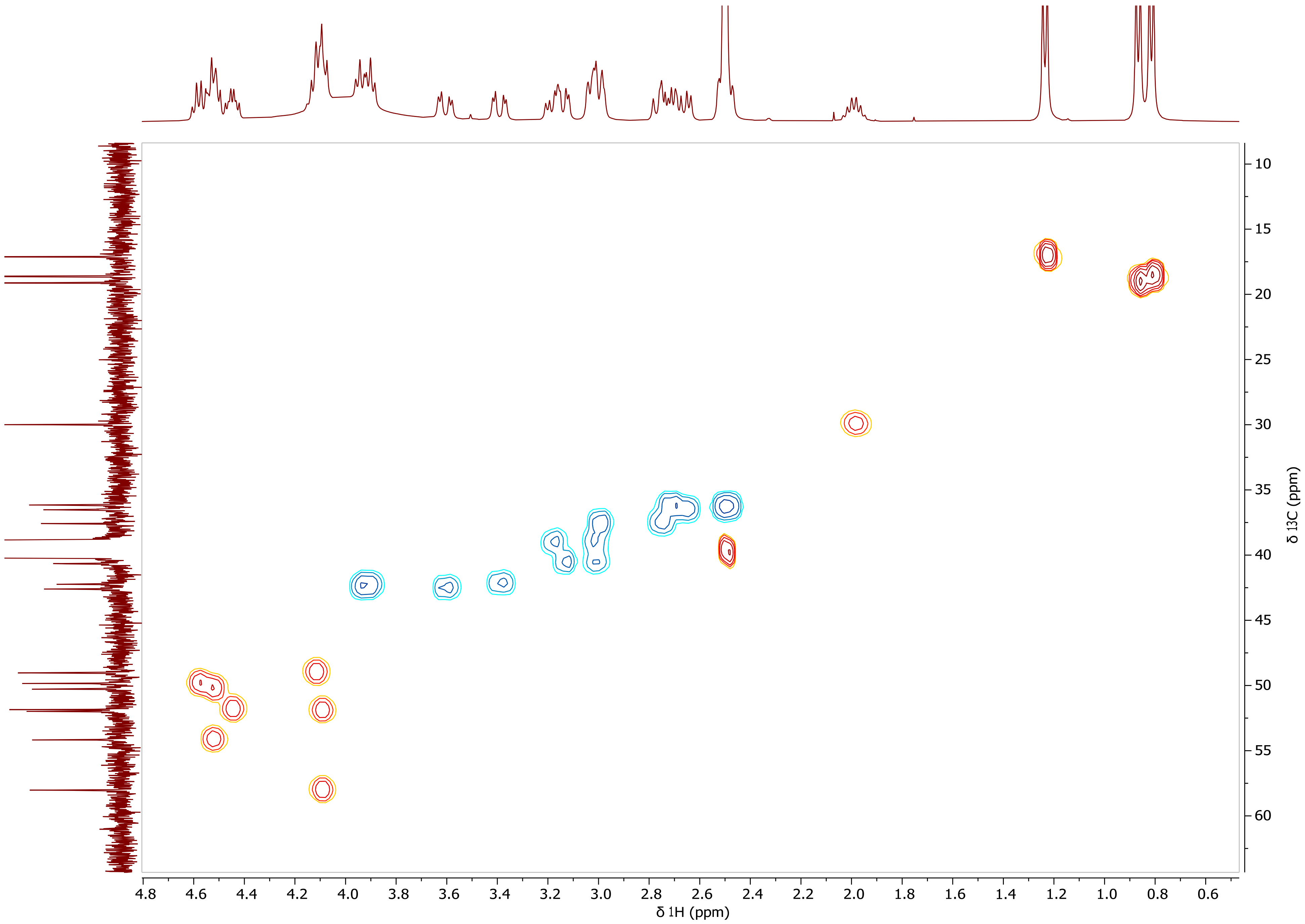

Figure S28: HSQC NMR (400 MHz) spectrum of LCP_O in DMSO-d_6

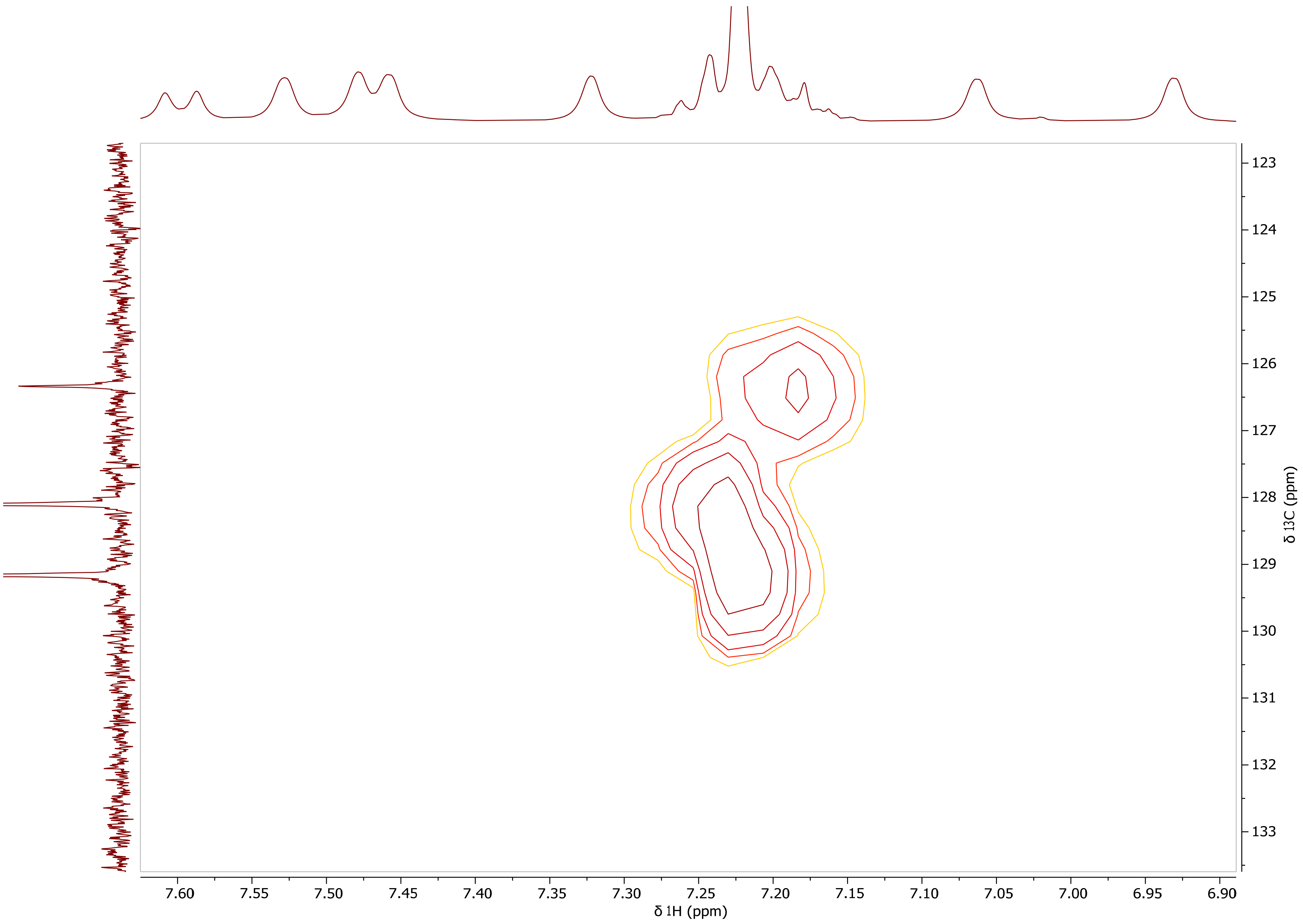

Figure S29: HSQC NMR (400 MHz) spectrum of LCP_O in DMSO-d_6

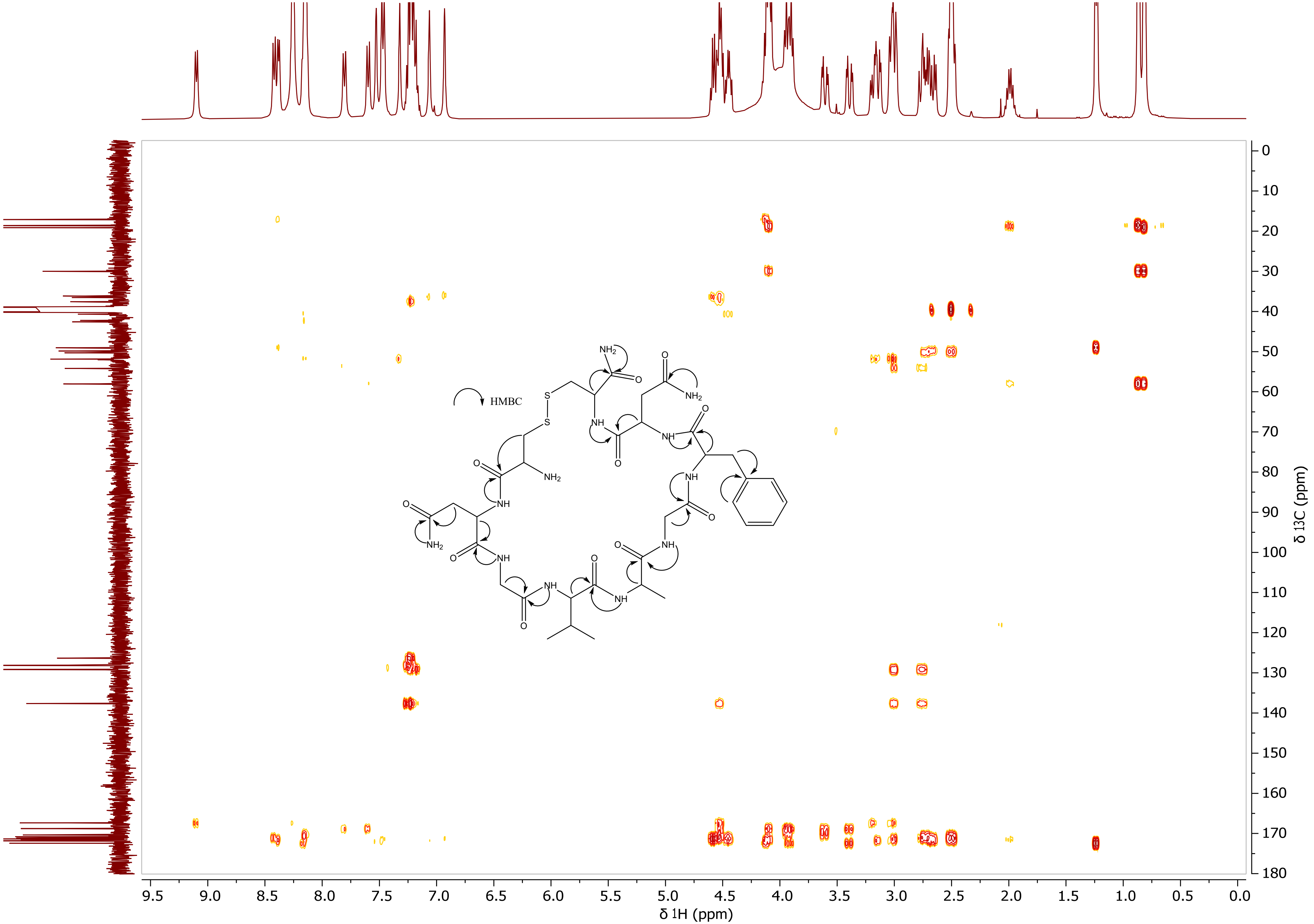

Figure S30: HMBC NMR (400 MHz) full spectrum of LCP_O in DMSO-d_6

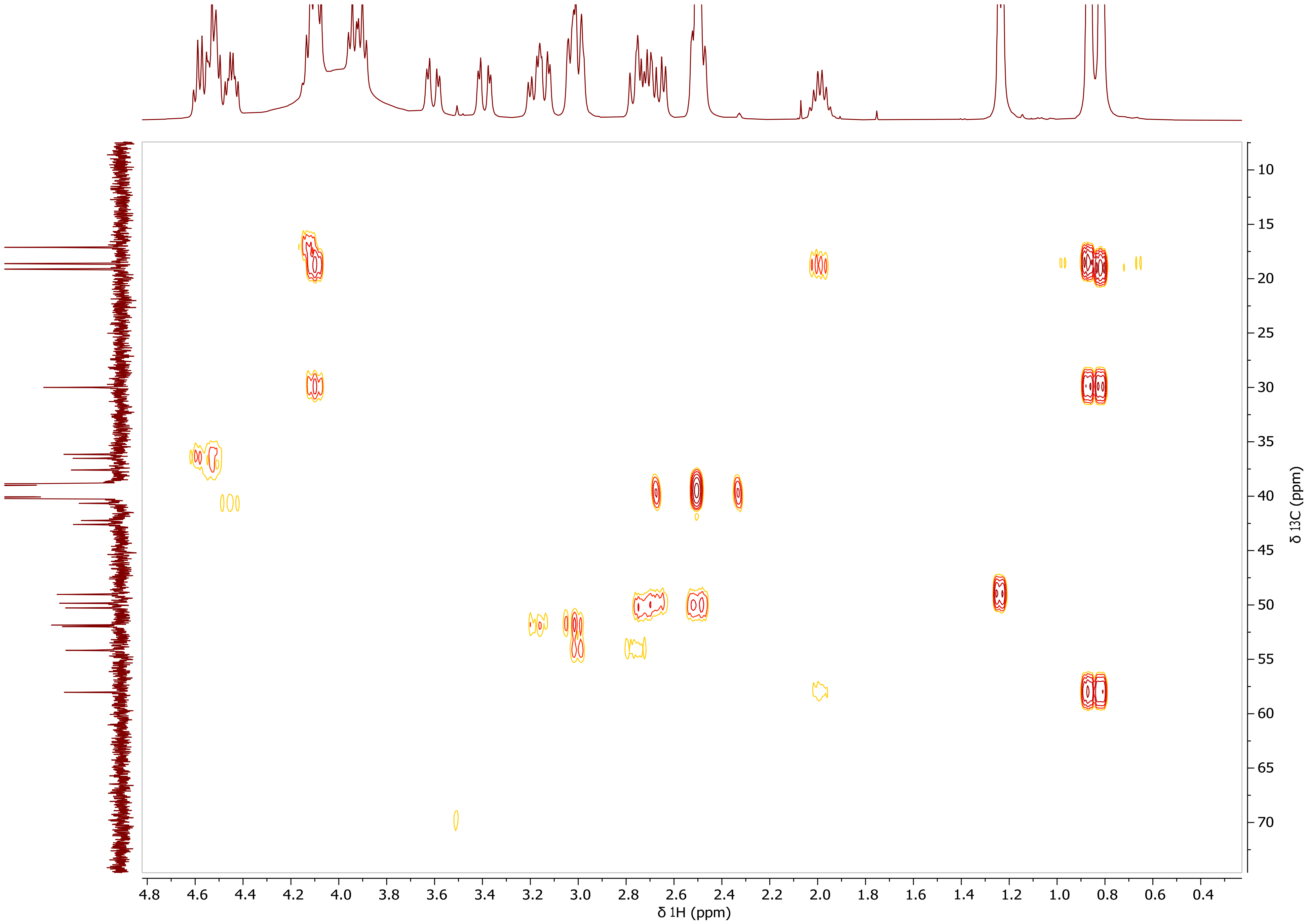

Figure S31: HMBC NMR (400 MHz) spectrum of LCP_O in DMSO-d_6

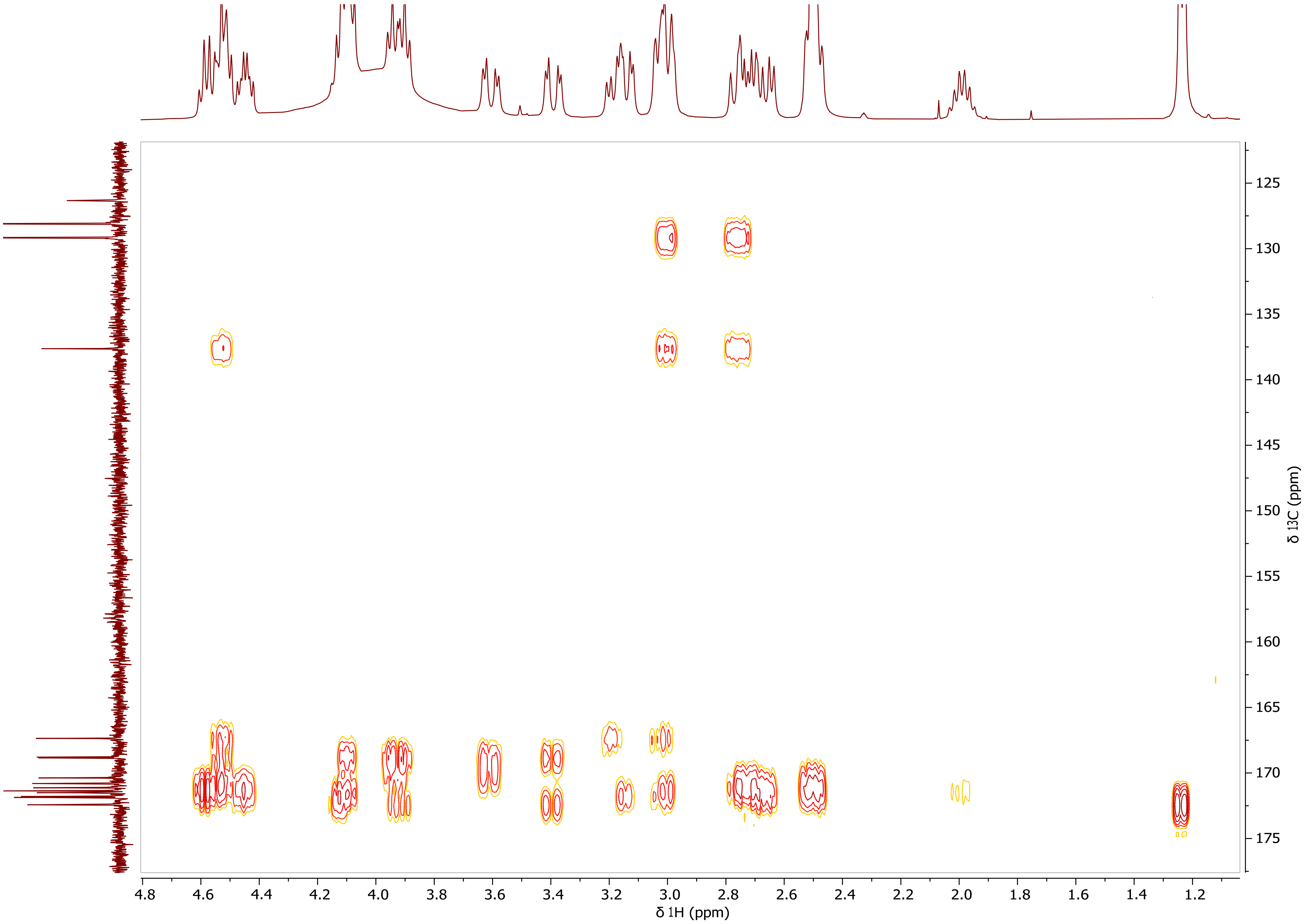

Figure S32: HMBC NMR (400 MHz) spectrum of LCP_O in DMSO-d_6

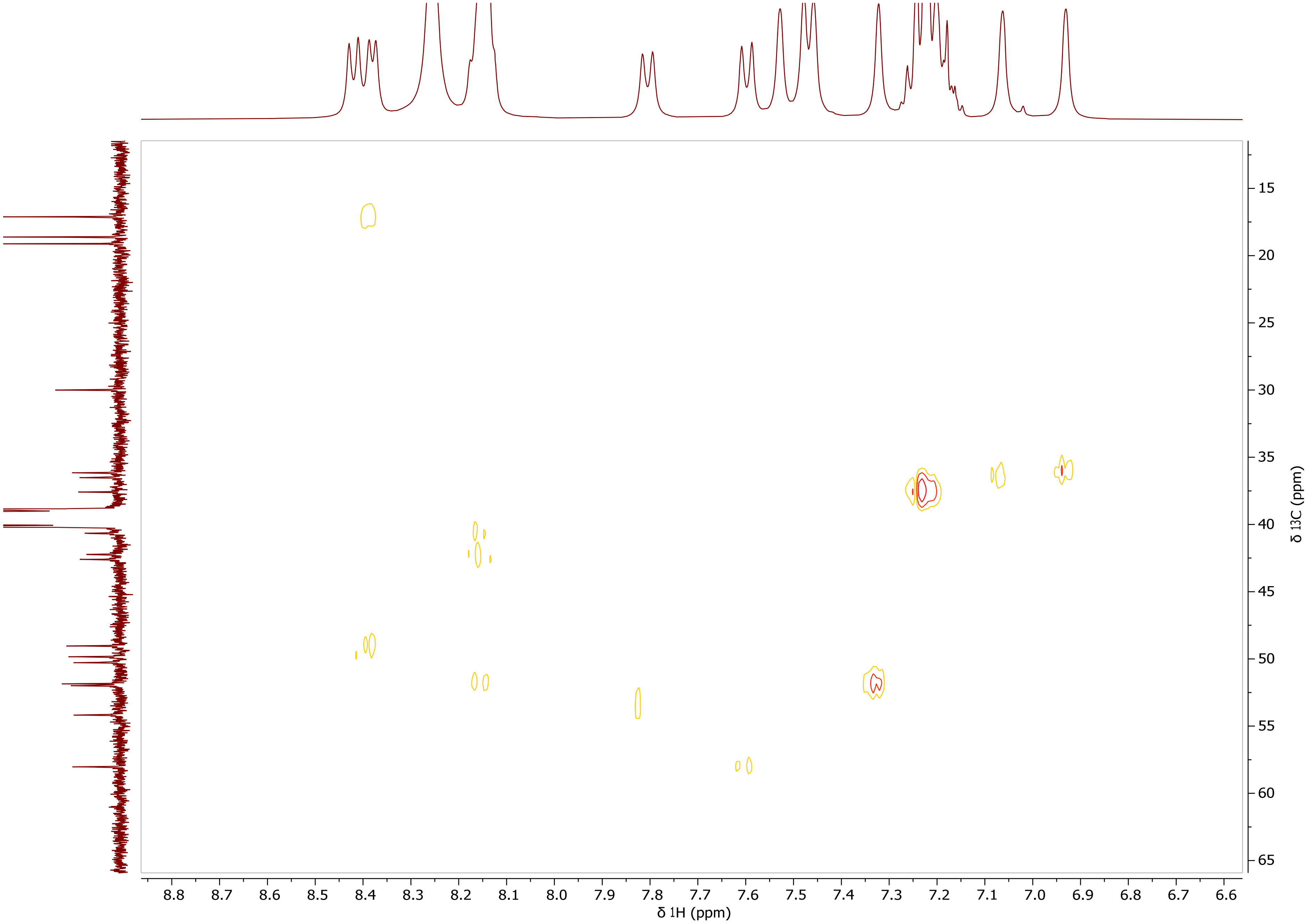

Figure S33: HMBC NMR (400 MHz) spectrum of LCP_O in DMSO-d_6

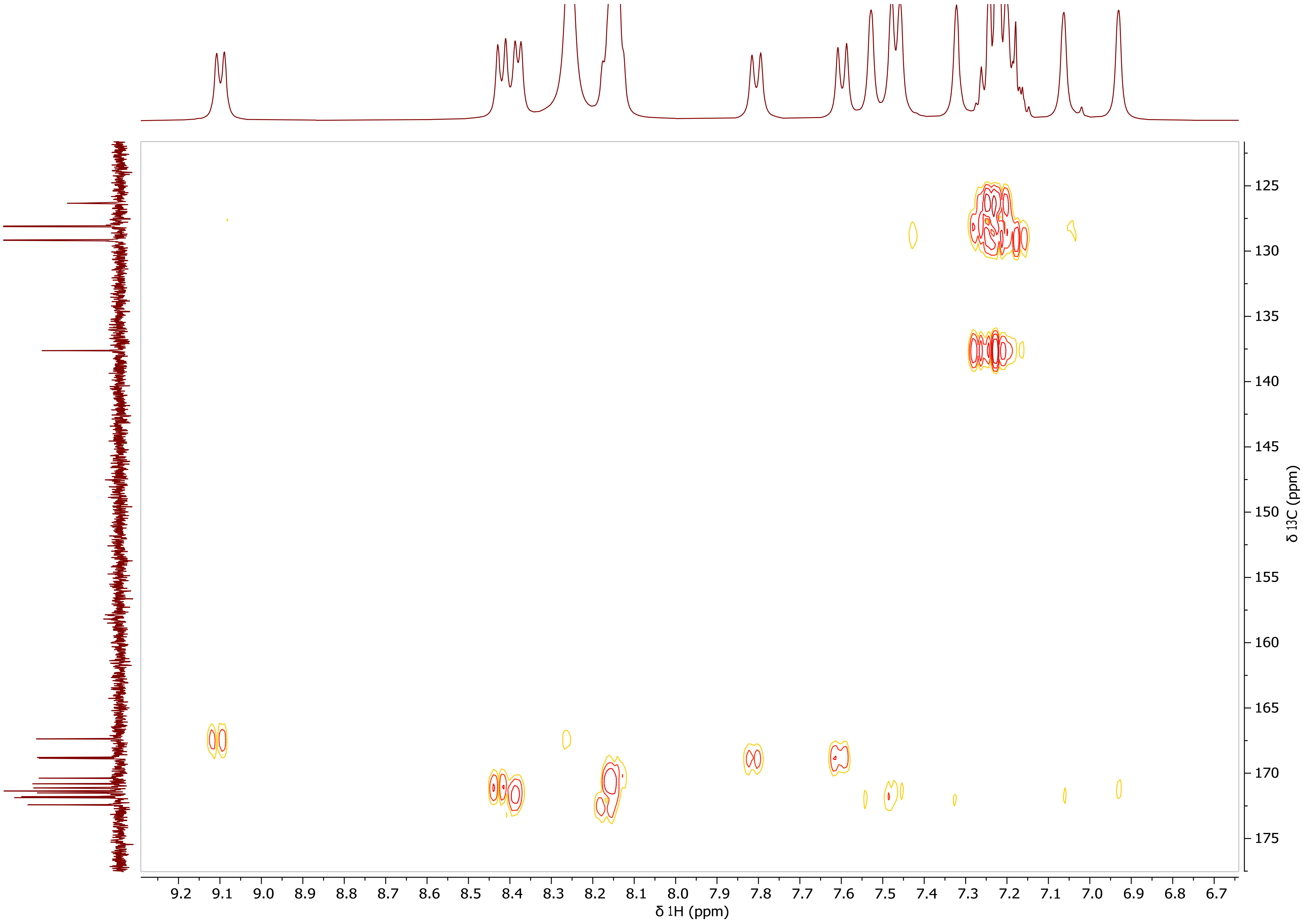

Figure S34: HMBC NMR (400 MHz) spectrum of LCP_O in DMSO-d_6

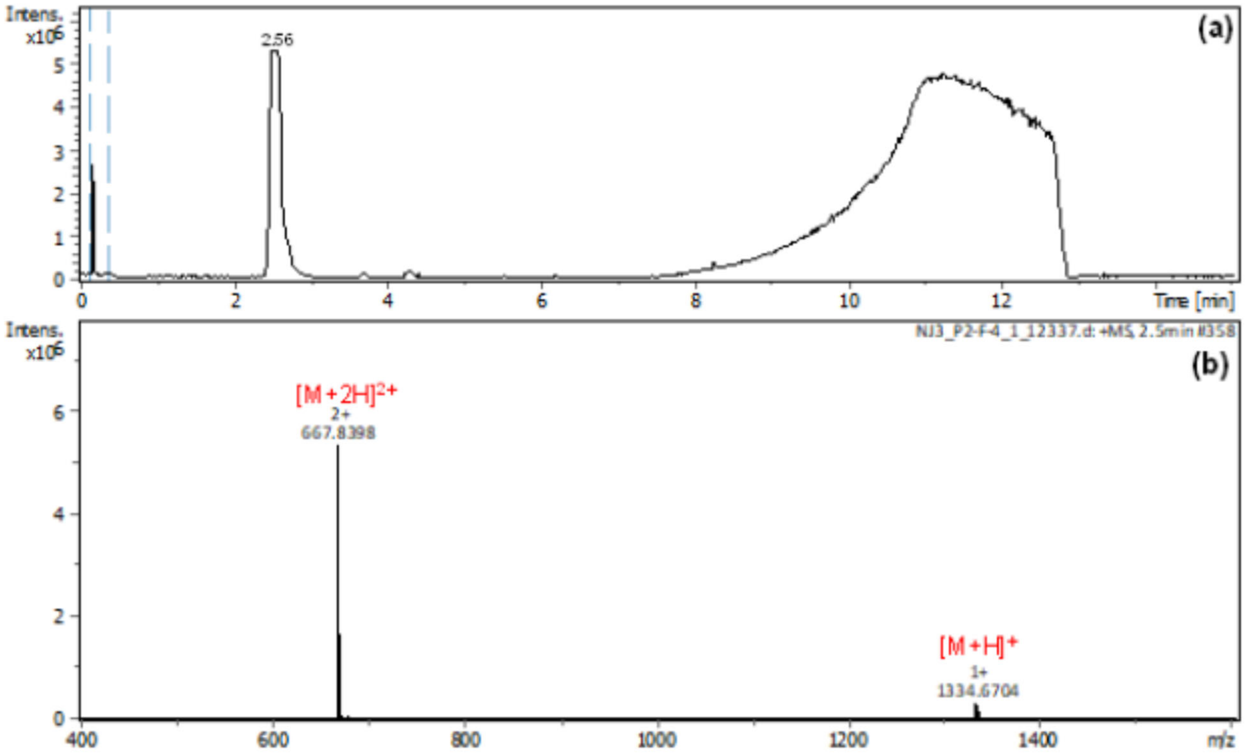

Figure S35: (a) LC HRMS Base peak chromatogram (BPC) of Pep42. (b) HRMS mass spectrum of 2.56 min peak showing $[M+H]^+$ (1334.6704 m/z) and $[M+2H]^{2+}$ (667.8398 m/z) peaks.

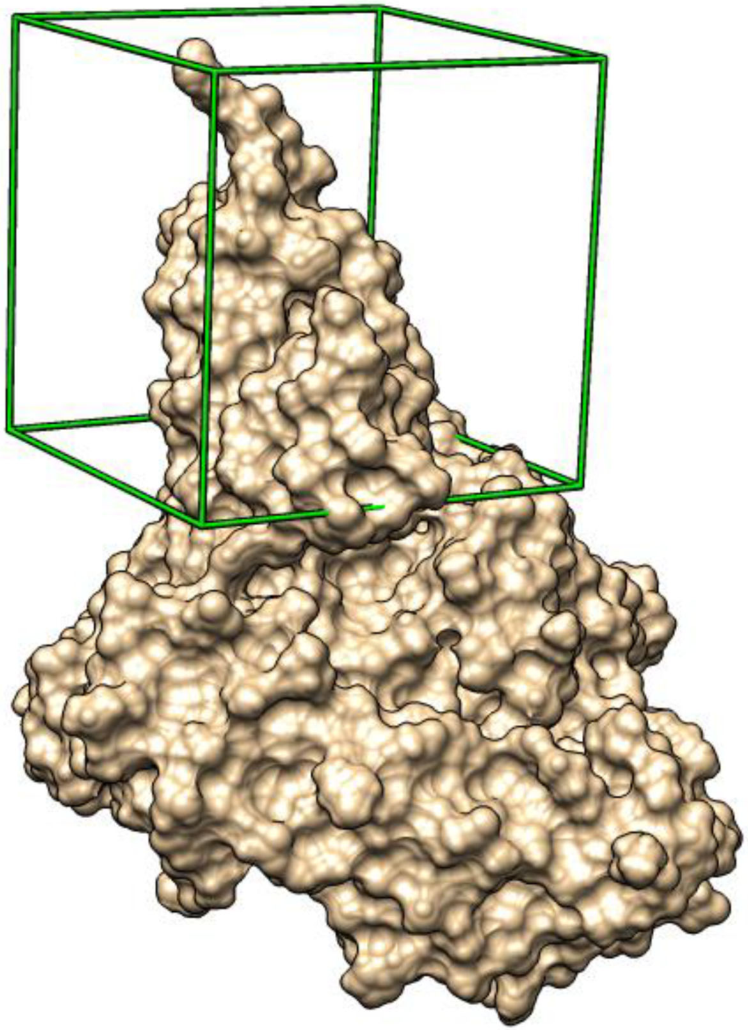

Figure S36: GRP78 structure (PDB 5E84, chain A) showing protein surface. The volume used in the docking calculations (40x40x40 Å) is shown in green, surrounding the SBDβ domain. As can be seen, all faces of the SBDβ were accessible to the cyclic peptide ligands during the docking calculations. Image created with UCSF Chimera 1.17.3.

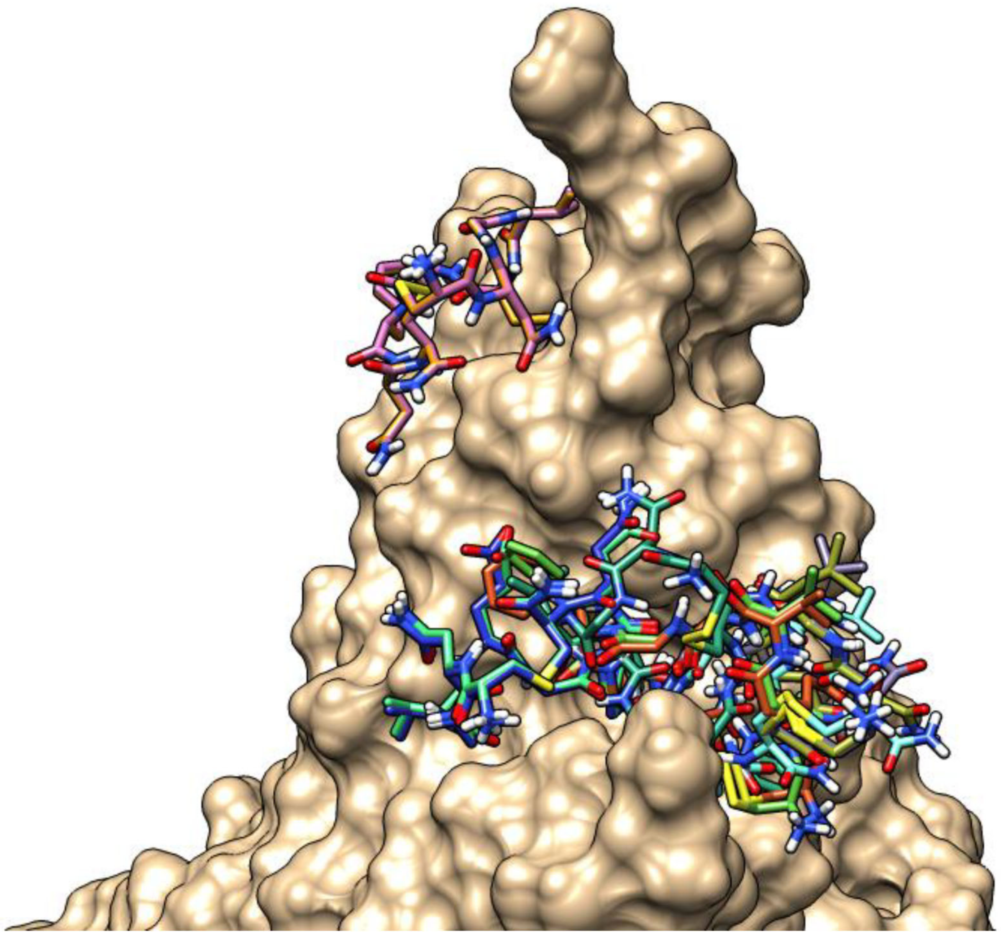

Figure S37: GRP78 structure (PDB 5E84, chain A) showing SBDβ surface with top ten docking poses for LCP_w. Image created with UCSF Chimera 1.17.3.

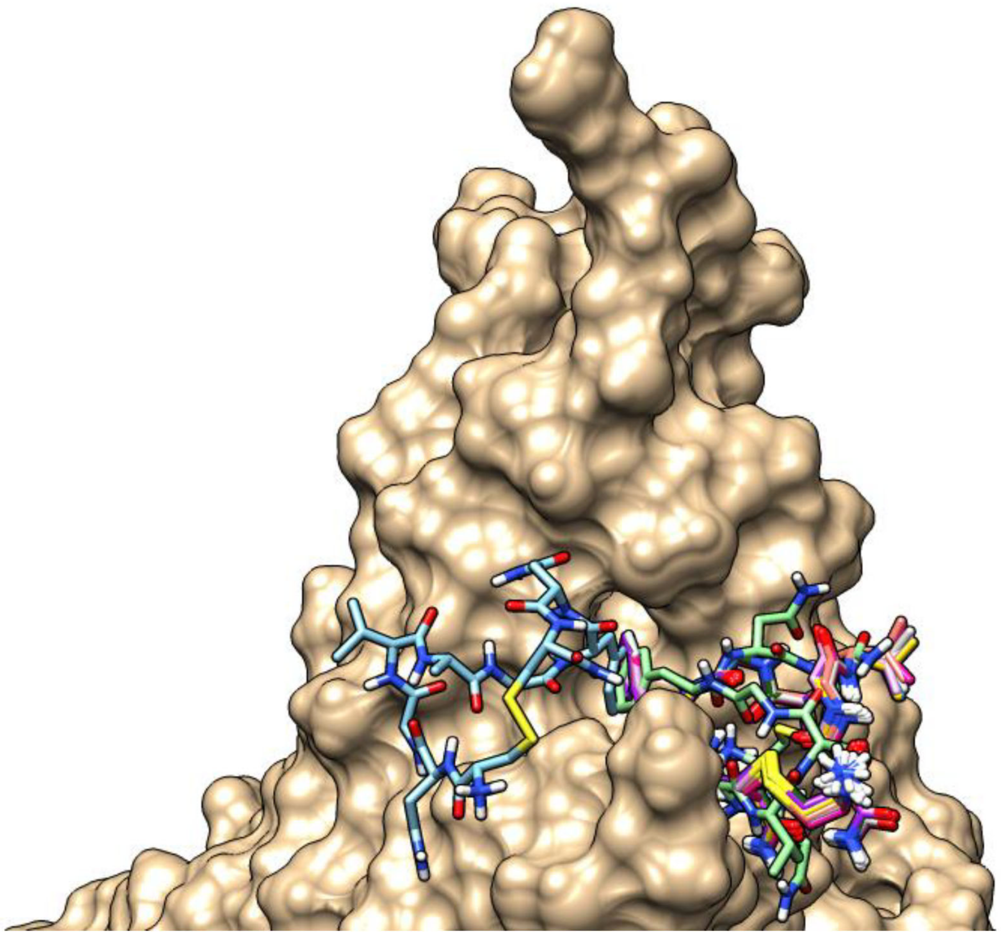

Figure S38: GRP78 structure (PDB 5E84, chain A) showing SBDβ surface with top ten docking poses for LCP_o. Image created with UCSF Chimera 1.17.3.

Table S3. GRP78 SBDβ residues involved in peptide binding in docking calculations. Residues shown in bold text are those previously identified by Ibrahim *et al*. in the analysis of the SARS-CoV-2 spike protein binding to GRP78.[17]

Hydrophobic Pocket	Surrounding Interacting Residues
**I426**	E427
**V429**	**T428**
**F451**	G430
V453	G431
**V457**	**V432**
**I459**	M433
	**T434**
	K435
	L436
	K447
	Q449
	I450
	**S452**
	G454
	T456
	T458
	K460
	T469
	T477
